# Interactions of amyloidogenic proteins with mitochondrial protein import machinery in aging-related neurodegenerative diseases

**DOI:** 10.3389/fphys.2023.1263420

**Published:** 2023-11-02

**Authors:** Ashley L. Reed, Wayne Mitchell, Andrei T. Alexandrescu, Nathan N. Alder

**Affiliations:** ^1^ Department of Molecular and Cell Biology, University of Connecticut, Storrs, CT, United States; ^2^ Department of Medicine, Brigham and Women’s Hospital, Harvard Medical School, Boston, MA, United States

**Keywords:** mitochondria, amyloids, neurodegeneration, protein import, cryptic targeting, targeting signals

## Abstract

Most mitochondrial proteins are targeted to the organelle by N-terminal mitochondrial targeting sequences (MTSs, or “presequences”) that are recognized by the import machinery and subsequently cleaved to yield the mature protein. MTSs do not have conserved amino acid compositions, but share common physicochemical properties, including the ability to form amphipathic α-helical structures enriched with basic and hydrophobic residues on alternating faces. The lack of strict sequence conservation implies that some polypeptides can be mistargeted to mitochondria, especially under cellular stress. The pathogenic accumulation of proteins within mitochondria is implicated in many aging-related neurodegenerative diseases, including Alzheimer’s, Parkinson’s, and Huntington’s diseases. Mechanistically, these diseases may originate in part from mitochondrial interactions with amyloid-β precursor protein (APP) or its cleavage product amyloid-β (Aβ), α-synuclein (α-syn), and mutant forms of huntingtin (mHtt), respectively, that are mediated in part through their associations with the mitochondrial protein import machinery. Emerging evidence suggests that these amyloidogenic proteins may present cryptic targeting signals that act as MTS mimetics and can be recognized by mitochondrial import receptors and transported into different mitochondrial compartments. Accumulation of these mistargeted proteins could overwhelm the import machinery and its associated quality control mechanisms, thereby contributing to neurological disease progression. Alternatively, the uptake of amyloidogenic proteins into mitochondria may be part of a protein quality control mechanism for clearance of cytotoxic proteins. Here we review the pathomechanisms of these diseases as they relate to mitochondrial protein import and effects on mitochondrial function, what features of APP/Aβ, α-syn and mHtt make them suitable substrates for the import machinery, and how this information can be leveraged for the development of therapeutic interventions.

## 1 Introduction

Alzheimer’s Disease (AD), Parkinson’s Disease (PD) and Huntington’s Disease (HD) are distinct neurodegenerative disorders that involve the progressive loss of neuronal structure and function. They are collectively classified as proteopathies, which involve severe disruption in cellular protein homeostasis (proteostasis) associated with protein misfolding as well as disruptions in protein processing and localization (Chowhan et al., 2015). Although the mechanistic causes of these diseases are incompletely understood, each is associated with the pathogenic accumulation of specific proteins. Amyloid-β (Aβ), the proteolytic product of amyloid-β precursor protein (APP), is associated with AD (Rocchi et al., 2003; Tcw and Goate, 2017); α-synuclein (α-syn) is associated with PD and other synucleinopathies (Stefanis, 2012); and expanded polyglutamine (poly-Q) repeats underpin a range of neurodegenerative disorders, including HD which is caused by mutant forms of huntingtin (mHtt) (Li et al., 1993; Schilling et al., 1995). Elucidating the mechanisms by which these proteins precipitate their respective pathogenic cascades will be essential in developing new treatments for their associated neurodegenerative diseases.

As protein deposition diseases, AD, PD and HD are associated with the conversion of soluble monomers or oligomers into highly organized insoluble fibrillar aggregates, called amyloid fibrils, that serve as their primary histopathological markers. Each of these neurodegenerative diseases is associated with a specific composition of protein deposits that target certain neuronal subpopulations within the central nervous system (CNS). For decades the dominant and unifying model to explain the etiology of these cerebral proteopathies has focused on aggregates of amyloid fibrils as the causative agents (Hardy and Higgins, 1992). However, in recent years this model has been challenged on two main fronts. First, there is accumulating evidence that monomers or small oligomers of Aβ, α-syn, and mHtt, rather than the large fibrils themselves, may in fact be the cytotoxic species; and second, the extent of fibrillization is not necessarily correlated with disease progression (Uddin et al., 2020; Wells et al., 2021).

These changes in perspective have been accompanied by an increasing recognition that mitochondrial dysfunction plays a key role in neurodegenerative diseases (Swerdlow et al., 2014). More specifically, APP/Aβ, α-syn, and mHtt have all been shown to negatively impact different mitochondrial functions and to accumulate within different mitochondrial subcompartments. Such observations raise the intriguing mechanistic question of how these amyloids target to mitochondria, given that mitochondria contain a network of machineries that are ostensibly designed to selectively import only polypeptides with defined functions in the organelle. Here we review emerging evidence that APP/Aβ, α-syn, and mHtt may contain “cryptic” sequences that mimic the classic N-terminal targeting information of mitochondrial proteins to interact with mitochondrial import complexes, the implications this may have for the role of mitochondrial dysfunction in neurodegeneration, and how such insights could inform the development of novel therapeutic strategies for AD, PD, and HD.

## 2 Mitochondria of the central nervous system

**FIGURE 1:**
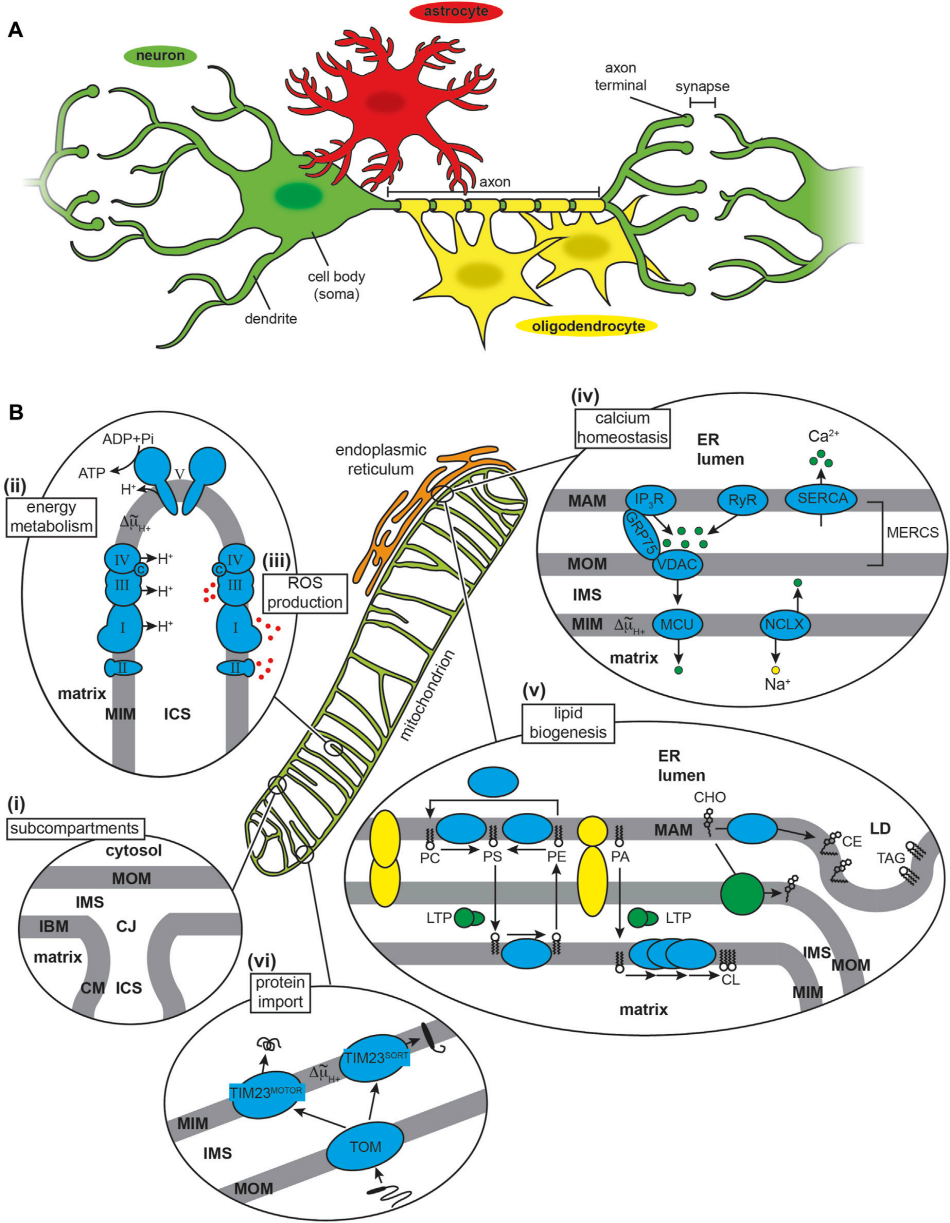
The CNS and mitochondria. **(A)** CNS cell types. The neuron (nerve cell, green) typically consists of a cell body (soma), multiple branching dendrites (afferent processes) that receive signals and transmit them to the cell body, and a single axon (efferent process) that forms an extended cable-like structure ending with axon terminals. Glial cells include astrocytes (red) and oligodendrocytes (yellow), amongst other cell types. **(B)** Mitochondria structure and major functions. (i) Subcompartments. Mitochondria have a two-membrane organization with a mitochondrial outer membrane (MOM) and inner membrane (MIM), the latter subdivided into an inner boundary membrane (IBM) closely appressed to the MOM, and the cristae membrane (CM). These membranes delineate the innermost matrix compartment from the intermembrane space (IMS) and intracristal space (ICS) that connect at the functional boundary of the crista junction (CJ). (ii) Energy metabolism. The OXPHOS machinery is parsed into complexes that generate an electrochemical proton potential (Δ^~^μ_H+_) across the CM (including respiratory complexes I, II, III and IV of the electron transport chain) and the F_1_F_O_ ATP synthase (complex V) that uses the energy of the proton gradient to drive ATP synthesis. (iii) ROS production. Mitochondrial complexes I, II and III generate superoxide (O_2_^.−^) from the one-electron reduction of O_2_, which can subsequently be catalytically dismutated to H_2_O_2_ (a potent signaling molecule) or converted to the cytotoxic hydroxyl radical (OH^·^). (iv) Calcium homeostasis. The sarco/endoplasmic reticulum acts as a Ca^2+^ repository based largely on Ca^2+^ influx from ATP-dependent SERCA pumps and Ca^2+^ efflux from channels including the inositol (1,4,5)-triphosphate (IP_3_R) receptor (tethered to the MOM by GRP75) and ryanodine receptor (RyR). Mitochondrial Ca^2+^ uptake across the MOM occurs through the voltage-dependent anion channel (VDAC) and across the MIM by the mitochondrial calcium uniporter (MCU), with efflux occurring primarily by the Na^+^/Ca^2+^ exchanger (NCLX). (v) Lipid biogenesis and trafficking. Lipid biosynthesis complexes (cyan) and lipid transport proteins (LTPs) mediate the production of phospholipids (PC, phosphatidylcholine; PS, phosphatidylserine; PE, phosphatidylethanolamine; PA, phosphatidic acid; and CL, cardiolipin) and cholesterol (CHO). The MAM also mediates formation of lipid droplet (LD), enriched in cholesterol esters (CE) and triacylglycerol (TAG). MAM-MOM tethering complexes are depicted in yellow. (vi) Protein import. Most mitochondrial proteins are nuclear encoded and imported from the cytosol. Nearly all proteins enter mitochondria through the TOM complex, and most are imported to the final destination by modular assemblies of the TIM complex (TIM23^MOTOR^ and TIM23^SORT^).

The CNS is composed of neurons and glial cells (Figure 1A). Neurons are morphologically complex cells that transmit information by receiving a stimulus at dendrites that is transferred to the cell body and then propagated as an action potential (an electrochemical impulse) along the axon. Glial cells provide physical and metabolic support to neurons and include several subtypes including astrocytes, which serve mainly to support neural function and signaling; oligodendrocytes, which form myelin sheaths around axons; and microglia, the immune cells of the CNS. Neurodegenerative disorders predominantly affect neurons in particular anatomical regions of the brain (Dugger and Dickson, 2017; Fu et al., 2018); however, glial cells are purported to also play a direct role in the pathomechanisms of these diseases (Gleichman and Carmichael, 2020).

Mitochondria are morphologically complex, bound by a mitochondrial outer (MOM) and inner (MIM) membrane that enclose the intermembrane space (IMS), intracristal space (ICS) and matrix aqueous compartments (Figure 1Bi). Mitochondria assume a particularly important role in the physiology of neurons and neuroglia, given the high metabolic and signaling activity of these cells (Kann and Kovacs, 2007). First, mitochondria of the CNS must ensure efficient energy metabolism (Figure 1Bii). The brain comprises only 2%–3% of the human body mass yet accounts for up to 20% of total energy expenditure (Rolfe and Brown, 1997). This high energy demand is due to processes that include the maintenance of ion gradients such as Na^+^/K^+^-ATPases and Ca^2+^-ATPases for neuronal excitability, as well as the synthesis, packaging, and cycling of neurotransmitters (Attwell and Laughlin, 2001). To meet this demand, most neuronal ATP is generated by mitochondrial oxidative phosphorylation (OXPHOS) fueled by glycolysis-derived pyruvate, although some glycolysis-derived ATP is utilized directly at nerve terminals during stress to sustain synaptic transmission (Tsacopoulos and Magistretti, 1996; Murali Mahadevan et al., 2021). The astrocyte-neuron lactatae shuttle model proposes that lactate produced by astrocytes is subsequently taken up by surrounding neurons to support high OXPHOS activity; however, the accuracy and relevance of this model has been questioned (Dienel, 2017). Second, mitochondria are the primary source of reactive oxygen species (ROS) in cells (Figure 1Biii), most of which are generated from the partial reduction of dioxygen (O_2_) by different enzyme complexes of the electron transport chian (ETC) (Halliwell, 1992). As in other tissues, ROS serve dual roles in CNS cells. On one hand, ROS are critical for signaling processes required for neuronal plasticity and network tuning, and on the other, mitochondrial dysfunction can lead to ROS overproduction and oxidative damage, which is positively associated with neurodegenerative disorders (Milton and Sweeney, 2012; Oswald et al., 2018). Other central functions of mitochondria involve their interactions with the endoplasmic reticulum (ER), specifically at specialized regions of the ER called the mitochondrial-associated membrane (MAM) that is tethered to the MOM at sites termed mitochondria-ER contact sites (MERCSs) (Aoyama-Ishiwatari and Hirabayashi, 2021; Sassano et al., 2022). One function of MERCSs is the regulation of Ca^2+^ homeostasis (Figure 1Biv). The ER serves as the primary Ca^2+^ storage organelle, balancing ion uptake by the sarco/endoplasmic reticulum Ca^2+^-ATPase (SERCA) pumps with transient release from channels that include the inositol 1,4,5-triphosphate receptor (IP_3_R) and the ryanodine receptor (RyR). Mitochondria serve as temporary stores of cellular Ca^2+^ (e.g., during Ca^2+^ transients that occur with action potentials in neurons), taking up ions through the β-barrel voltage-dependent anion channel (VDAC) in the MOM and the mitochondrial calcium uniporter (MCU) of the MIM. Ca^2+^ dyshomeostasis is a central feature of neurodegenerative diseases (Kolobkova et al., 2017; McDaid et al., 2020; Xu et al., 2022a). MERCSs also regulate lipid biosynthesis (Figure 1Bv) by serving as platforms for the non-vesicular trafficking of phospholipids and cholesterol and lipid droplet formation (Giordano, 2018; Benador et al., 2019). Defects in lipid metabolism are also a central feature of neurodegeneration, much of it attributable to alterations at the MAM-mitochondria interface (Block et al., 2010; Alecu and Bennett, 2019; Yin, 2023). Finally, mitochondria contain protein import machinery for the biogenesis of nuclear-encoded proteins (Figure 1Bvi). The role of the import machinery in mediating mitochondrial interactions with amyloidogenic proteins is the focus of this review.

Mitochondria are highly dynamic organelles, constantly undergoing growth, fission into fragments balanced by fusion into interconnected networks, and selective degradation of dysfunctional organelles by mitophagy (Yapa et al., 2021). Furthermore, neuronal mitochondria are distributed to match the local metabolic and signaling requirements of the somatic, dendritic, axonal, and synaptic regions, a process governed by anterograde and retrograde trafficking (Mandal and Drerup, 2019). Mitochondrial biogenesis requires the regulated import of proteins into the organelle to accommodate growth and replacement of damaged proteins to maintain an adequate population of healthy mitochondria.

Mitochondria also have a specialized lipid composition (Claypool and Koehler, 2012; Poulaki and Giannouli, 2022). The glycerophospholipid cardiolipin is of particular relevance because it is unique to mitochondria. Cardiolipin has an unusual structure, with a two-phosphate headgroup that imparts a strong negative charge to the membrane surface and four acyl tails, creating a molecular geometry that affects lipid packing and stabilizes local membrane curvature (Ikon and Ryan, 2017). Cardiolipin is primarily localized to the MIM, where it accounts for approximately 20 mol% of total phospholipid content, and is less abundant in the MOM, where it makes up less than 5 mol% of phospholipids. Externalization of cardiolipin from the MIM to the MOM can occur with cellular stress, which can serve as a signal for selective mitochondrial autophagy (mitophagy) or programmed cell death (apoptosis) (Li et al., 2015).

A key question surrounding neurodegeneration is why neurons are particularly vulnerable to proteostatic imbalance. The answer is manifold: because neurons are terminally differentiated and non-proliferative cells, they cannot rely on asymmetric mitosis to purge aggregated proteins and must therefore rely on robust proteostatic quality control machinery that can fail with age (Heydari et al., 1994; Conconi et al., 1996; Taylor and Dillin, 2011); because neurons are structurally polarized with long processes, the clearance of protein aggregates from distal parts of the cell is energetically costly and prone to dysregulation (Guo et al., 2020); and because neurons have such specialized functional and metabolic demands (e.g., maintenance of ion gradients, calcium regulation, and neurotransmitter cycling), there is a small energetic margin of error to spare in the face of proteostatic stress, particularly with age-related decreases in energy metabolism (Blaszczyk, 2020).

## 3 Mitochondrial protein import and the proteostatic network

### 3.1 The mitochondrial protein import and quality control machinery

**FIGURE 2:**
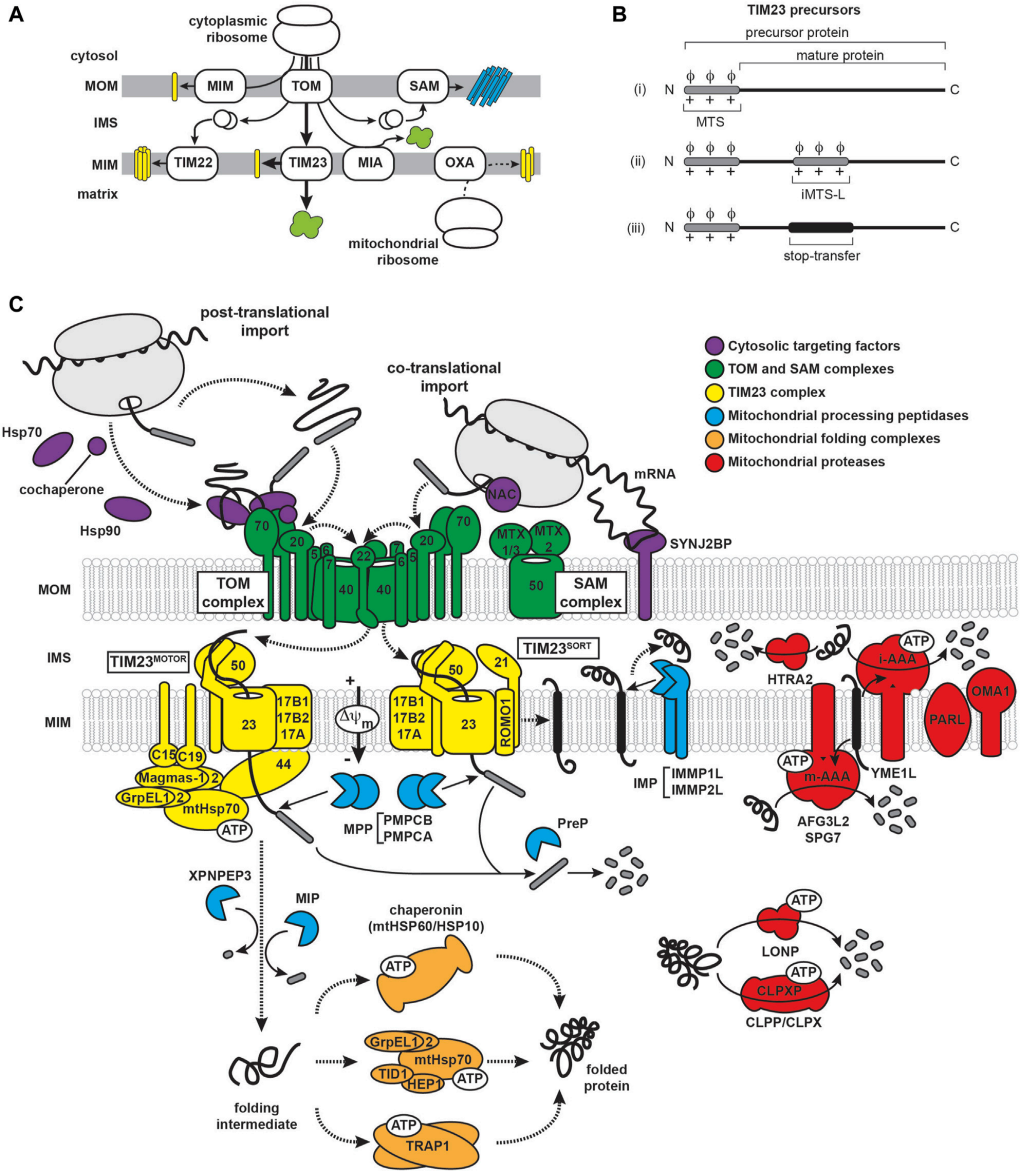
Mitochondrial import and proteostatic control. **(A)** Trafficking routes of mitochondrial proteins. Mitochondrial proteins include soluble proteins of the IMS/ICS and matrix (green), α-helical membrane proteins of the MOM and MIM (yellow) and β-barrel proteins of the MOM (cyan). Trafficking routes are depicted for nuclear-encoded proteins (solid lines) and mitochondrial-encoded proteins (dashed line). The major protein transport complexes include the translocase of the outer membrane (TOM) complex, the mitochondrial import (MIM) complex, the sorting and assembly machinery (SAM) complex, translocases of the inner membrane 22 and 23 (TIM22 and TIM23, respectively), the mitochondrial IMS import and assembly (MIA) complex, and the oxidase assembly (OXA) insertase. The trafficking of MTS-containing precursors via the TIM23 pathway is denoted by the thick arrows. **(B)** Targeting and topogenic sequences of TIM23 substrates. The MTSs are depicted in gray as containing basic (+) and hydrophobic (ϕ) residues. The stop-transfer segment is depicted in black. **(C)** The mammalian TIM23 import, processing, and quality control machinery. Components of the biogenesis machinery are categorized as: (i) chaperones that regulate the targeting of precursors from the cytosol to the mitochondrion (violet), (ii) outer membrane (TOM and SAM) complexes (green), (iii) TIM23^MOTOR^ and TIM23^SORT^ complexes (yellow), (iv) proteases that process TIM23 substrates (cyan), (v) matrix chaperones that mediate the folding of TIM23 substrates (orange), and (vi) proteases that mediate the degradation of mitochondrial proteins (red). See Supplementary Table S1 for a listing of all relevant proteins.

The biogenesis and steady-state function of mitochondria require a highly regulated system of protein import, sorting, assembly, and quality control (Figure 2; Supplementary Table S1). The human mitochondrial proteome consists of approximately 1200–1500 individual proteins (Morgenstern et al., 2021; Rath et al., 2021). Being semi-autonomous organelles, mitochondria have the genome (mitochondrial DNA) and the biosynthetic machinery (e.g., mitochondrial ribosomes, RNA/DNA polymerases and tRNAs) to synthesize a handful of their resident proteins, which in humans includes 13 subunits of OXPHOS complexes I, III, IV and V. All other mitochondrial proteins are encoded in nuclear DNA, synthesized on cytosolic ribosomes, and subsequently imported into mitochondria. Multiple pathways exist for the targeting and sorting of nuclear-encoded proteins to the proper mitochondrial membrane or aqueous subcompartment (Busch et al., 2023) (Figure 2A). These proteins are synthesized with mitochondria targeting information encoded in the polypeptide sequence itself, which must be recognized by the dedicated import complexes that direct them to their correct destinations. The majority (about two-thirds) of mitochondria-targeted proteins are imported via the translocase of the mitochondrial inner membrane 23 (TIM23) pathway, which mediates the translocation of soluble proteins into the matrix as well as the integration of membrane proteins into the MIM (Sinha et al., 2014). In this section, we summarize our current understanding of TIM23-based protein biogenesis and the associated mitochondrial proteostasis machinery.

#### 3.1.1 TIM23 presequences

A key defining feature of TIM23 substrates is an amino-terminal targeting signal termed the mitochondrial targeting sequence (MTS), or “presequence”. These proteins are synthesized as so-called precursors that are recognized by receptors of the mitochondrial import machinery and typically have their respective MTSs cleaved to yield the mature form of the protein upon reaching their destination. The MTS signals lack sequence conservation, are highly variable in length (ranging from about 15 to 60 amino acids), and are unstructured in aqueous solution. However, they all share a capacity to form amphipathic α-helices with one face enriched in hydrophobic residues and the other enriched in basic residues (mostly Arg). This is coupled with a near absence of acidic residues, resulting in a net charge between +3 and +6 (Roise et al., 1986; Roise et al., 1988; Roise and Schatz, 1988; Pak and Weiner, 1990; Vogtle et al., 2009; Calvo et al., 2017). TIM23-targeted precursors have a diversity of targeting elements (Figure 2B): (i) those containing only the N-terminal MTS followed by a soluble mature protein, (ii) those with MTS-like structures (iMTS-Ls) in the mature protein that bind mitochondrial receptors and enhance import efficiency and kinetics (Backes et al., 2018; Hansen et al., 2018), and (iii) those with a bipartite signal sequence that, in addition to the MTS, contain a topogenic (membrane-active) hydrophobic stop-transfer sequence that partitions into the MIM as a transmembrane segment (Glick et al., 1992). It should also be noted that MTS processing is not a strict feature of TIM23-mediated import, as some TIM23 precursors are not processed following translocation and some retain import competence even with their MTSs deleted (Longen et al., 2014; Woellhaf et al., 2014; Weill et al., 2018). Importantly, the variability of the sequence and the locations of MTS and MTS-like sequences may explain the ability of some amyloidogenic polypeptides to present cryptic TIM23 targeting signals, as we discuss later.

#### 3.1.2 Cytosolic trafficking of TIM23 substrates

The biogenesis of TIM23 substrates begins on cytosolic ribosomes and is regulated by several molecular chaperones and piloting factors (Figure 2C, violet). The targeting of these proteins to mitochondria mostly proceeds post-translationally, whereby the polypeptide is completely synthesized and released from the ribosome before engaging the mitochondrial import machinery. In this case, ATP-dependent cytosolic heat shock proteins (HSPs) of the HSP70 (Deshaies et al., 1988; Murakami et al., 1988; Terada et al., 1995; Endo et al., 1996) and HSP90 families (Young et al., 2003; Fan et al., 2006) may bind precursors at different stages to prevent their aggregation and maintain them in partially unfolded states (Becker et al., 2019; Avendano-Monsalve et al., 2020), which is particularly important for precursor proteins with transmembrane segments (Claros et al., 1995). The HSP70/90 chaperones undergo ATPase cycles that are allosterically coupled to substrate binding and release (Rutledge et al., 2022). HSP70s inhibit folding of their client substrates by extensively interacting with low specificity at binding motifs of short hydrophobic segments flanked by charged residues (Rudiger et al., 1997). By comparison, HSP90s have more extended binding sites that recognize later-folding intermediates (Karagoz and Rudiger, 2015). The activity of HSP70s is modulated by co-chaperones that regulate ATP turnover and substrate specificity (Moran Luengo et al., 2019). HSP70 co-chaperones include J domain (HSP40) proteins, which stimulate ATP hydrolysis, and nucleotide exchange factors (NEFs), which promote exchange of bound ADP for ATP (Kampinga and Craig, 2010). Multiple J domain co-chaperones, including DNAJA1, 2, and 4 in mammals and Djp1, Ydj1 and Sis1 in yeast, have been implicated in precursor protein targeting to mammalian mitochondria (Bhangoo et al., 2007; Papic et al., 2013; Jores et al., 2018).

Alternatively, the targeting of TIM23 substrates can proceed co-translationally, wherein the polypeptide engages the mitochondrial import machinery while it is still being translated on the ribosome (Avendano-Monsalve et al., 2020; Lenkiewicz et al., 2021). The existence of cotranslational import in both yeast and mammalian mitochondria is supported by evidence of mitochondria-bound ribosomes and polysomes that is promoted, for example, following treatment with the translation elongation inhibitor cycloheximide (Crowley and Payne, 1998; Williams et al., 2014; Gold et al., 2017). Other studies similarly support cotranslational targeting of different TIM23 substrates (Ahmed et al., 2006; Yogev et al., 2007). Although mitochondria do not appear to have a dedicated cotranslational targeting route (for instance, analogous to the signal recognition particle-mediated pathway of the ER (Akopian et al., 2013)), there are systems in place for promoting cotranslational mitochondrial import under certain conditions. For example, mRNAs encoding mitochondria-targeted proteins are enriched at the mitochondrial surface (Egea et al., 1997; Matsumoto et al., 2012; Williams et al., 2014; Fazal et al., 2019; Kuzniewska et al., 2020), and stabilized by MOM-localized RNA binding proteins Puf3 in yeast (Wang et al., 2018) and perhaps SYNJ2BP in mammals (Qin et al., 2021). Additionally, a translation stimulator at the mitochondrial surface, the MDI-Larp complex, was shown to enhance protein synthesis in the vicinity of import complexes in *Drosophila* (Zhang et al., 2016). Finally, mitochondria-targeted proteins can be recognized by the heterodimeric nascent polypeptide-associated complex (NAC) (Beatrix et al., 2000), which simultaneously binds ribosomes and emerging nascent chains and may promote cotranslational targeting to mitochondria (Wiedmann et al., 1994; George et al., 1998; George et al., 2002; del Alamo et al., 2011; Gamerdinger et al., 2019). In yeast, NAC binds to the SAM complex subunit Sam37 (Ponce-Rojas et al., 2017; Avendano-Monsalve et al., 2022) and the MOM protein OM14 (Lesnik et al., 2014); whether NAC engages homologous proteins in mammalian mitochondria remains an open question. NAC may play a special role in amyloidogenic diseases, as it has recently been shown to suppress aggregation of poly-Q expanded proteins (Shen K. et al., 2019). The critical point is that regardless of whether precursor substrates are imported co- or post-translationally, the targeting system is designed to maintain the substrate in an unfolded state in order to preserve its import competence.

#### 3.1.3 Structure and function of the TOM complex

**FIGURE 3:**
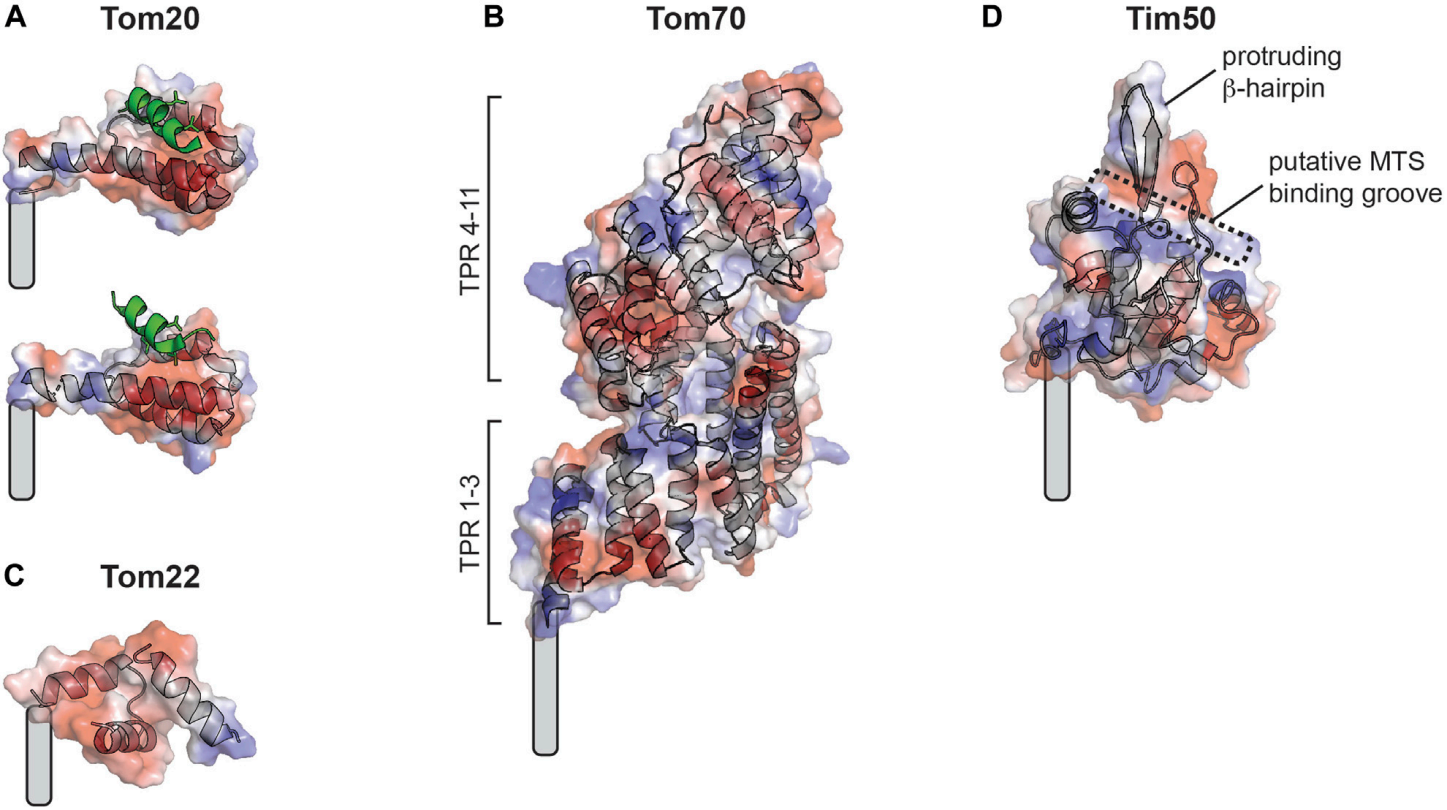
Mitochondrial protein import receptors of known structure. Soluble receptor domains are shown as cartoon traces superimposed with electrostatic surfaces (red, acidic; blue, basic; white, neutral). Grey curved rectangles show approximate positions of transmembrane segment attachment. **(A)** Rat Tom20 receptor from crystal structure with disulfide-bound MTS (green) showing two different binding states resolved by distinct MTS-receptor chemical tethering strategies (the so-called A-linker [PDB 2V1T], above; and the Y-linker [PDB 2V1S], below). **(B)** Yeast Tom70 receptor from crystal structure (PDB 2GW1). **(C)** Human Tom22 receptor from cryo-EM structure (PDB 7VDD). **(D)** Tim50 receptor (homology model of human receptor based on yeast Tim50 core domain) from crystal structure (PDB 3QLE), showing the predicted MTS binding pocket and protruding β-hairpin.

The translocase of the outer mitochondrial membrane (TOM) complex of the MOM serves as the entry site for all TIM23 substrates into mitochondria (Figure 2C, green). This complex contains seven different subunits: the Tom40 β-barrel channel that serves as the aqueous transmembrane conduit for precursors; small TOM proteins (Tom5, Tom6, Tom7) that regulate TOM complex assembly; and several receptors, including Tom22, Tom20 and Tom70 (Pitt and Buchanan, 2021; Araiso and Endo, 2022). The core complex appears to form as equi-stoichiometric assemblies of Tom40/22/5/6/7 that can arrange as dimeric or higher order structures (Kunkele et al., 1998; Model et al., 2008; Mager et al., 2010; Shiota et al., 2015; Bausewein et al., 2017; Sakaue et al., 2019); in contrast, the Tom20 and Tom70 receptors appear to be more loosely bound. Thus, these receptors may instead assemble with the TOM complex in an on-demand basis depending on the presence of substrate (Dekker et al., 1998; Bhagawati et al., 2021). Recent cryo-EM structures of the TOM complex in yeast (Araiso et al., 2019; Tucker and Park, 2019) and human (Wang et al., 2020a; Guan et al., 2021; Su et al., 2022) have shed light on the structural interactions among TOM subunits and how precursor proteins are recognized and translocated. For example, the TOM receptors play complementary and partially overlapping roles in the recognition of MTS-containing proteins. Tom20, Tom70, and Tom22 all have receptor domains containing tetratricopeptide repeat (TPR) motifs that mediate protein interactions (Zeytuni and Zarivach, 2012) and appear to have general protein chaperone function in addition to acting as precursor receptors (Yano et al., 2004; Yamamoto et al., 2009). Tom20 serves as the general receptor for preproteins and is paradigmatic for MTS-receptor interactions because it is the only mitochondrial receptor for which high-resolution structural information is available in the MTS peptide-bound state (Abe et al., 2000; Saitoh et al., 2007; Saitoh et al., 2011). The Tom20 cytosolic C-terminal receptor domain contains two helix-turn-helix motifs that define a single prototypical TPR motif with an embedded nonpolar patch flanked by two acidic regions and a region rich in Gln residues (Figure 3A). The binding groove of Tom20 is shallow and short, accommodating only about eight residues of the MTS. The Tom20 recognition motif within MTSs is ϕχχϕϕ, where ϕ is a nonpolar residue and χ is any residue. This relatively nonspecific recognition motif enables a dynamic, weak-affinity and multi-mode interaction with the substrate, dominated by nonpolar contacts (Muto et al., 2001; Obita et al., 2003). Tom70, by comparison, has a much larger receptor domain containing 11 TPR motifs that are divided into N- and C-terminal parts (Wu and Sha, 2006) (Figure 3B). The N-terminal region of Tom70 contains a homodimerization interface and forms a clamp-like region (TPR motifs 1–3) that binds Hsp70 and Hsp90 chaperones, perhaps serving as a co-chaperone for the transfer of Hsp70/90-bound precursors to the TOM complex (Young et al., 2003; Wu and Sha, 2006). This function underscores its recently discovered role in recruiting chaperones to the mitochondrial surface (Backes et al., 2021). The C-terminal region of Tom70 (TPR motifs 4–11) forms a large pocket that likely binds polytopic membrane precursor proteins destined for the TIM22 pathway (Wiedemann et al., 2001; Rehling et al., 2003), and may bind targeting signals of TIM23 substrates as well (Hines et al., 1990; Hines and Schatz, 1993; Wu and Sha, 2006; Melin et al., 2015). The central receptor Tom22 regulates TOM complex assembly, and unlike the primary Tom20 and Tom70 receptors, it is tightly bound to the TOM complex. Additionally, Tom22 features receptor domains on both the cytosol-facing (Figure 3C) and IMS-facing sides of the MOM (Bolliger et al., 1995; Honlinger et al., 1995; Dekker et al., 1998; van Wilpe et al., 1999; Yamano et al., 2008). While Tom22 and Tom20 have similar substrate profiles (Mayer et al., 1995; Yamano et al., 2008), the Tom20 receptor mediates hydrophobic interactions with the MTS, whereas Tom22 interactions are dominated by electrostatic attraction between the basic face of the MTS and the partially disordered acidic Tom22 binding pocket (Kiebler et al., 1993). However, the strict requirement of these negatively charged residues has been questioned (Nargang et al., 1998). The dominant model describing how precursor proteins traverse the membrane through the TOM complex is by an “acid chain” of negatively charged patches on TOM subunits that guide the positively charged MTS. By this model, Tom5 and Tom22 make up an acidic pathway on the cytosolic face of the MOM (the *cis* site) (Bolliger et al., 1995; Dietmeier et al., 1997; Schatz, 1997; Komiya et al., 1998), the precursor moves through the Tom40 pore guided by acidic residues on the interior wall of the β-barrel (Suzuki et al., 2000; Gabriel et al., 2003; Shiota et al., 2015), and the precursor then binds sites on the IMS side of the MOM comprised of Tom22, Tom40 and Tom7 (the *trans* site) (Court et al., 1996; Moczko et al., 1997; Rapaport et al., 1997; Kanamori et al., 1999; Esaki et al., 2004). The selective positioning of acidic binding sites with increasing affinity for the MTS from the cytosolic to the IMS sites drives the vectorial movement of the precursor through the TOM complex.

#### 3.1.4 Structure and function of the TIM23 complex

The TIM23 complex is the dedicated machinery of the MIM that mediates the import and sorting of all MTS-containing precursor proteins (Genge and Mokranjac, 2021) (Figure 2, yellow). The general organization of TIM23 is evolutionarily conserved; however, in comparison with the more fully understood yeast complex, human TIM23 forms multiple functionally distinct complexes containing alternate subunit isoforms (Sinha et al., 2014; Pfanner et al., 2019). The core TIM23 complex in humans contains the channel forming Tim23 and Tim17A/B1/B2 isoforms that assemble to make an aqueous conduit across the MIM for the passage of preproteins (Bauer et al., 1999; Moro et al., 1999; Martinez-Caballero et al., 2007; Demishtein-Zohary et al., 2017; Matta et al., 2017). It also contains Tim50, which serves as the main receptor for MTS-bearing precursors (Geissler et al., 2002; Yamamoto et al., 2002; Mokranjac et al., 2003a) and, in mammals, acts as a broad specificity phosphatase (Guo et al., 2004; Chaudhuri et al., 2021). The conserved core domain of the Tim50 receptor contains a putative MTS binding groove lined with negatively charged and hydrophobic residues as well as a prominent β-hairpin (Qian et al., 2011) (Figure 3D). The N-terminal extension of the Tim23 channel is an intrinsically disordered region that specifically interacts with Tim50 near the β-hairpin (Geissler et al., 2002; Yamamoto et al., 2002; Mokranjac et al., 2003a; Meinecke et al., 2006; Alder et al., 2008a; Gevorkyan-Airapetov et al., 2009; Mokranjac et al., 2009; Tamura et al., 2009; Qian et al., 2011; Schulz et al., 2011; Lytovchenko et al., 2013; Malhotra et al., 2017; Dayan et al., 2019; Gunsel et al., 2020). This interaction between the disordered N-terminal extension of Tim23 and Tim50 maintains the Tim23 channel in a quiescent, dimeric state that preserves the transmembrane potential (Δψ_m_) across the MIM (Bauer et al., 1996; Meinecke et al., 2006; Alder et al., 2008a).

To deliver precursors to their correct destination, the compositionally dynamic TIM23 complex forms two different assemblies adapted to the Tim23/17/50 core. The TIM23^MOTOR^ complex mediates the translocation of soluble precursors into the matrix (Mokranjac, 2020), a process that requires the recruitment of a matrix-localized molecular motor system (the presequence translocase-associated motor, or PAM complex) that includes: the central subunit mitochondrial Hsp70 (mtHsp70, also called mortalin), which serves as the ATP-driven molecular motor (Goswami et al., 2010; Esfahanian et al., 2023); Tim44, which anchors mtHsp70 to the TIM23^MOTOR^ complex (Kronidou et al., 1994; Schneider et al., 1994; Silva et al., 2004); and co-chaperones that modulate the ATPase activity of mtHsp70. The latter include the GrpE-Like 1 and 2 (GrpEL1 and GrpEL2) NEFs (Schneider et al., 1996; Naylor et al., 1998; Srivastava et al., 2017), the paralogous DnaJC15 (C15) and DnaJC19 (C19) that are homologous to the yeast J-protein Pam18/Tim14 (Silva et al., 2003; Mokranjac et al., 2003b; Richter-Dennerlein et al., 2014), and isoforms of the mitochondria-associated granulocyte-macrophage colony stimulating factor (GM-CSF) signaling molecule (Magmas-1 and 2) that are orthologs of the yeast J-like protein Pam16/Tim16 (Elsner et al., 2009; Sinha et al., 2010; Waingankar and Silva, 2021). By contrast, the TIM23^SORT^ complex mediates the lateral sorting of membrane-directed precursor proteins into the MIM. This sorting complex lacks the PAM motor and recruits additional membrane subunits. These include Tim21, which makes specific contacts with Tim23 and Tim50 (Tamura et al., 2009; Lytovchenko et al., 2013; Bajaj et al., 2014) and mediates the assembly of membrane-bound subunits of respiratory complex IV (Mick et al., 2012; Richter-Dennerlein et al., 2016), and ROMO1, a subunit homologous to yeast Mgr2 that promotes the interaction of Tim21 with the TIM23^SORT^ complex and is specifically required for the import of mitochondrial proteases (Ieva et al., 2014; Richter et al., 2019; Matta et al., 2020). Apropos of these proposed discrete TIM23^SORT^ and TIM23^MOTOR^ models for MIM integration and matrix import, it should be noted that other experimental results support a model in which TIM23 is a single structural entity that is actively remodeled to support translocation or integration depending on substrate availability instead of existing in two disparate states (Popov-Celeketic et al., 2008).

The import of MTS-containing substrates by the TIM23 complex is a multistep, energy-requiring process. The transfer of precursor proteins to the TIM23 complex is facilitated by the formation of a TOM-TIM23 supercomplex that is stabilized by interactions of the IMS-facing *trans* site of the TOM complex and the IMS-facing regions of Tim21, Tim50, and Tim23 subunits of the TIM23 complex (Dekker et al., 1997; Chacinska et al., 2003; Mokranjac et al., 2005; Albrecht et al., 2006; van der Laan et al., 2006; Chacinska et al., 2010; Shiota et al., 2011; Gold et al., 2014), which have been verified primarily by crosslinking experiments done with yeast models and with purified proteins (Chacinska et al., 2005; Mokranjac et al., 2005; Albrecht et al., 2006; Tamura et al., 2009; Shiota et al., 2011; Bajaj et al., 2014; Waegemann et al., 2015; Araiso et al., 2019; Gunsel et al., 2020). Upon emerging from the TOM complex, the MTS first binds the Tim50 receptor, thereby displacing receptor interactions with the Tim23 N-terminus and Tim21 and altering the TOM-TIM23 association (Geissler et al., 2002; Yamamoto et al., 2002; Mokranjac et al., 2003a; Mokranjac et al., 2009; Marom et al., 2011a; Shiota et al., 2011; Lytovchenko et al., 2013; Waegemann et al., 2015). The MTS then binds the now-exposed Tim23 N-terminus (Bauer et al., 1996; de la Cruz et al., 2010; Marom et al., 2011a; Lytovchenko et al., 2013) and is directed to the Tim23 channel which, like Tom40, has specific residues along the channel lumen that interact with substrates (Alder et al., 2008b; Denkert et al., 2017). The dynamic Tim23 channel undergoes conformational alterations in response to substrate and changes in the Δψ_m_ (Popov-Celeketic et al., 2008; Malhotra et al., 2013) and the presence of the MTS activates the Tim23 channel gating (Bauer et al., 1996; Truscott et al., 2001). The basic MTS is electrophoretically pulled toward the negatively charged matrix through the activated Tim23 channel (Martin et al., 1991), with unidirectional movement imparted by increasing binding affinity between the MTS and Tim23, Tim50, and Tim44 (Marom et al., 2011b). Soluble precursor proteins are then translocated completely into the matrix by the TIM23^MOTOR^ complex, whereby the ATPase activity of mtHsp70, modulated by co-chaperones DnaJC15/19 and Magmas-1/2, pulls the substrate by a Brownian ratchet or active pulling mechanism (Mokranjac et al., 2003a; Mokranjac, 2020; Silva et al., 2003; Mokranjac et al., 2003b; Truscott et al., 2003). By contrast, when a hydrophobic stop-transfer sequence is detected on the substrate, translocation stalls, the complex recruits subunits of the TIM23^SORT^ complex, and the nonpolar segment partitions laterally into the MIM as an α-helical transmembrane segment in a manner driven by the Δψ_m_ (Gartner et al., 1995; Gruhler et al., 1997) and mediated by the ROMO1 and Tim21 gatekeepers (van der Laan et al., 2006; Mick et al., 2012; Ieva et al., 2014; Richter-Dennerlein et al., 2016; Richter et al., 2019; Lee et al., 2020). Notably, an alternative structure-based model of TIM23 function suggests that instead of forming an aqueous channel, Tim23 and Tim17 together form lipid-exposed cavities that provide a protein translocation pathway (Sim et al., 2023), consistent with evidence that TIM23 precursors are translocated across the MIM at the Tim17-bilayer interface rather than via a channel defined by Tim23 (Fielden et al., 2023).

#### 3.1.5 Processing and quality control of TIM23 substrates

During import, precursor proteins are selectively processed to their mature forms by a set of mitochondria-localized processing proteases (Gomez-Fabra Gala and Vogtle, 2021; Kunova et al., 2022) (Figure 2C, cyan). The vast majority of TIM23 complex substrates are processed in a way that removes the N-terminal targeting sequences (Vogtle et al., 2009). The main protease is the matrix-localized mitochondrial processing peptidase (MPP), a metalloendopeptidase that forms a dimeric complex (PMPCA and PMPCB subunits in human) (Taylor et al., 2001). MPP cleaves at defined recognition sites (predominantly with the scissile bond two or three residues C-terminal to an Arg residue (Calvo et al., 2017)), thereby releasing the MTS which is subsequently degraded by the presequence protease (PreP) (Alikhani et al., 2011a; Kucukkose et al., 2021). Following MTS cleavage, some matrix-targeted precursors require additional maturation steps at the new N-terminus that involve the removal of either a single residue (mediated by the XPNPEP3 protease (Singh et al., 2017)) or an octapeptide (mediated by the MIP protease (Vogtle et al., 2011)), both of which remove destabilizing N-terminal residues to increase protein half-life (Vogtle et al., 2009; Varshavsky, 2011). Additionally, some TIM23-targeted substrates integrated into the MIM are processed by the inner membrane peptidase IMP (IMMP1L and IMMP2L in human), which releases a soluble IMS-facing domain of the imported protein as the mature, functional form (Nunnari et al., 1993).

To ensure the proper folding of newly-imported proteins, mitochondria contain two main chaperone systems in the matrix (Figure 2C, orange). The mitochondrial Hsp60/Hsp10 chaperonin complex, a homolog of the bacterial GroEL/GroES chaperonin, sequesters unfolded or kinetically-trapped folding intermediates inside an Anfinsen cage-like cavity and undergoes ATPase-driven structural changes to release properly folded proteins (Cheng et al., 1989; Ellis, 1996; Nielsen and Cowan, 1998; Apetri and Horwich, 2008; Chakraborty et al., 2010; Nisemblat et al., 2015). In addition, a soluble complex of mtHsp70 (mortalin) resides in the matrix (Horst et al., 1997; Havalova et al., 2021), where it performs its protein folding functions with three co-chaperones that have been identified in human: the Hsp70-escort protein 1 (HEP1) and J-domain protein tumorous imaginal disc protein 1 (TID-1), which regulate ATPase activity of mtHsp70, and the NEFs GrpEL1/2 (Sichting et al., 2005; Zhai et al., 2008; Iosefson et al., 2012; Dores-Silva et al., 2013; Havalova et al., 2021). These main matrix chaperone systems are supplemented in mammals by the HSP90 paralog TRAP1, which performs diverse functions including acting as a late-stage folding chaperone for mitochondrial matrix proteins (Joshi et al., 2022).

In addition, mitochondria contain several proteases for the degradation of misfolded and damaged proteins (Gomez-Fabra Gala and Vogtle, 2021) (Figure 2C, red). In human mitochondria, four main ATP-fueled proteases of the AAA+ (ATPases associated with diverse cellular activities) superfamily are responsible for the surveillance and clearance of proteins. These include the MIM-bound metalloproteases *m*-AAA (homo-oligomers of AFG3L2 or hetero-oligomers of AFG3L2 and SPG7 with catalytic domains facing the matrix) and *i*-AAA (composed of YME1L1 with catalytic domains facing the IMS), both of which can extract and break down MIM proteins (Opalinska and Janska, 2018). Additionally, the matrix contains soluble AAA+ serine proteases. LONP1 is a homohexameric assembly that serves as the central quality control protease in the matrix, degrading misfolded and damaged proteins (Szczepanowska and Trifunovic, 2022) in addition to promoting protein folding by cooperating with mtHsp70 (Shin et al., 2021). The CLPXP complex, on the other hand, is a heterooligomeric protease assembly involved in diverse functions including mitoribosome and OXPHOS maintenance (Szczepanowska and Trifunovic, 2022). The HTRA2 (high temperature requirement) soluble serine protease of the IMS is involved in caspase-dependent apoptosis and has been implicated in PD progression (Vande Walle et al., 2008). Finally, there are additional MIM-bound proteases, including PARL and OMA1, that have a more specific set of substrate proteins that regulate mitochondrial dynamics, mitophagy, and stress responses (Pellegrini and Scorrano, 2007; Jiang et al., 2014). As discussed below, the quality control machinery for newly imported proteins may also be involved in stress responses involving amyloidogenic proteins.

### 3.2 Amyloid misfolding and cellular proteostasis

**FIGURE 4:**
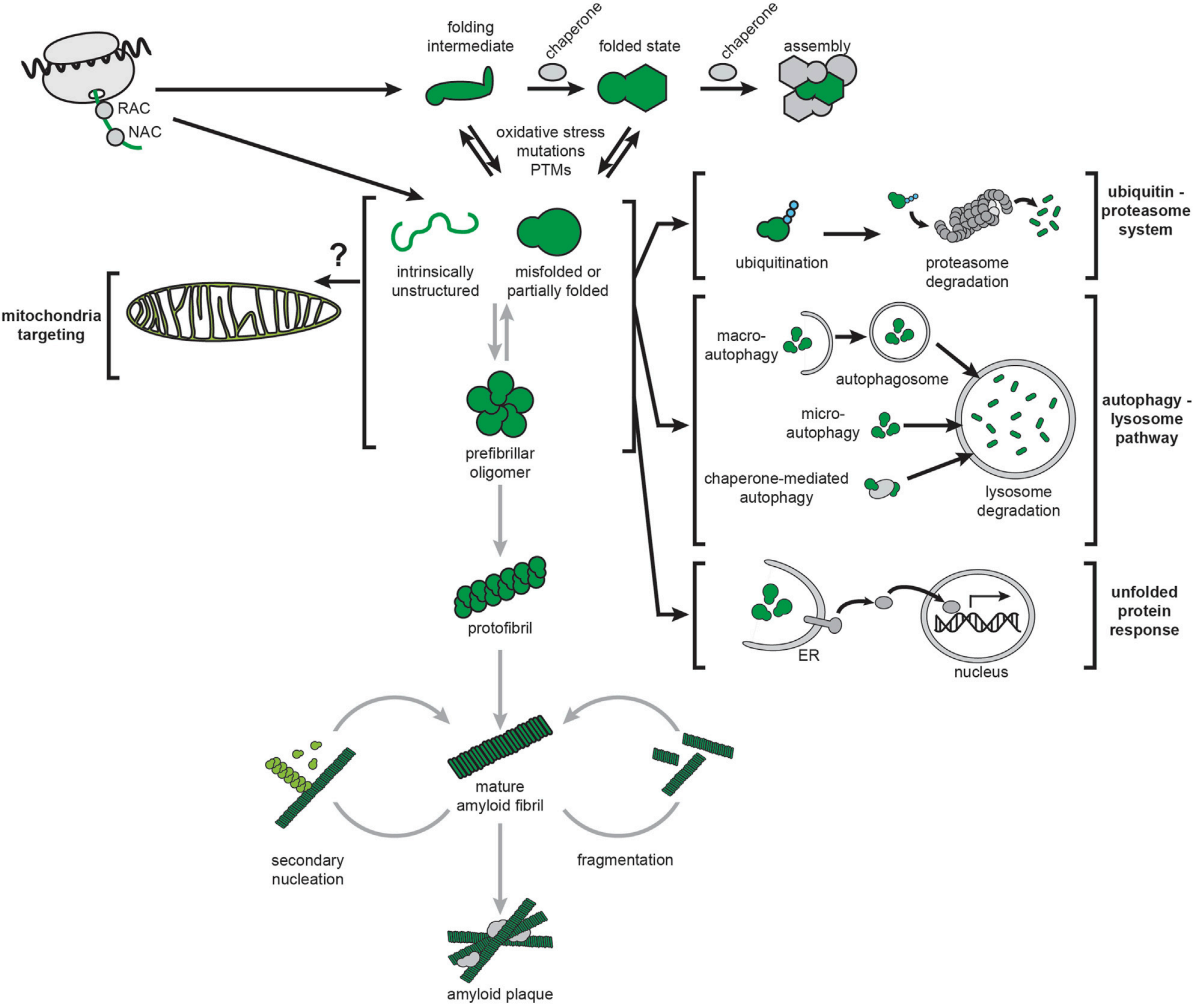
Amyloids and cellular proteostasis. The proteostasis network of mammalian cells is depicted by black arrows. Chaperones that guide cotranslational folding on the ribosome include the nascent polypeptide-associated complex (NAC) and the ribosome-associated complex (RAC). Additional chaperones guide monomer folding and assembly into complexes. The ubiquitin-proteasome system (UPS) targets misfolded proteins, or proteins that are no longer needed, by ubiquitination and digestion by the proteasome. The autophagy-lysosome pathway (ALP) targets misfolded proteins (and larger structures such as large aggregates and organelles) by delivering them to the lysosome via distinct pathways (macroautophagy, microautophagy and chaperone-mediated autophagy). Protein unfolding stress can activate complex signal transduction pathways leading to gene expression changes for restoring protein homeostasis, most notably the ER-based unfolded protein response (UPR^ER^). Amyloidogenesis, depicted by gray arrows, can initiate from intrinsically disordered or misfolded structures and proceed by stepwise formation of oligomers, protofibrils, and finally mature fibrils. It can also be accelerated by secondary nucleation and fragmentation of existing fibrils.

Cellular proteostasis involves the regulation of all stages of the protein life cycle (Labbadia and Morimoto, 2015; Klaips et al., 2018): molecular chaperones guide cotranslational folding of nascent chains during ribosomal synthesis and promote protein folding and assembly of oligomeric complexes, degradation mechanisms such as the ubiquitin-proteasome system (UPS) and the autophagy-lysosome pathway (ALP) remove misfolded and aggregated proteins, and stress response pathways respond to protein folding stress, such as the ER-based unfolded protein response (UPR^ER^) (Figure 4, black arrows). All three of these protein quality control processes are implicated in neurodegenerative diseases (Scheper and Hoozemans, 2015; Park et al., 2020; Schmidt et al., 2021).

AD, PD, and HD are proteostatic diseases associated with the misfolding of Aβ, α-syn, and mHtt, respectively, in a process that leads to the formation of amyloid fibrils (Figure 4, grey arrows). This process can begin with precursors that include intrinsically disordered peptides such as Aβ and α-syn, or with partially and misfolded globular proteins. The kinetics of amyloid fibrillization have a marked dependence on protein concentration, as well as factors that affect the tendency for polypeptides to self-associate, including mutations, post-translational modifications (PTMs), cofactors, and oxidative stress (Alexandrescu, 2005; Rezaei-Ghaleh et al., 2016; Hu et al., 2019; Lontay et al., 2020; Chiki et al., 2021; Gottlieb et al., 2021; He et al., 2021; McGlinchey et al., 2021). Fibrillization follows nucleation kinetics, accounting for the “seeding” properties of amyloids (Chiti and Dobson, 2017; Almeida and Brito, 2020). Nucleation can occur through primary or *de novo* processes, or secondary mechanisms whereby mature fibril surfaces act as templates for new fibril growth (Ferrone et al., 1985; Padrick and Miranker, 2002; Patil et al., 2011; Tornquist et al., 2018). Amyloid fibrils share a common structural property of protein association through a cross-β spine motif, stabilized by intermolecular hydrogen-bonded parallel β-sheet layers arranged perpendicular to the long axis of the fibril (Nelson et al., 2005; Chiti and Dobson, 2017; Almeida and Brito, 2020). They also share similar morphologies, often being microns long with widths of ∼10 nm and a twisting repeat of ∼100 nm (Dobson, 2003; Qiang et al., 2017).

Amyloidogenic aggregates have historically provided the primary histopathological markers of AD, PD, and HD. These aggregates are generally termed “amyloid fibrils” when formed extracellularly and ‘inclusions’ when formed intracellularly. The hallmarks of AD are extracellular plaques composed primarily of Aβ peptides and intracellular neurofibrillary tangles enriched in hyperphosphorylated variants of the protein tau (Zheng and Koo, 2011). PD is characterized by cytoplasmic aggregates called Lewy bodies and inclusions called Lewy neurites, of which α-syn is the main component (Braak et al., 1999). Intracellular accumulation of mHtt into amyloid-like inclusion bodies is a primary feature of HD (Yamamoto et al., 2000; Arrasate et al., 2004).

**FIGURE 5:**
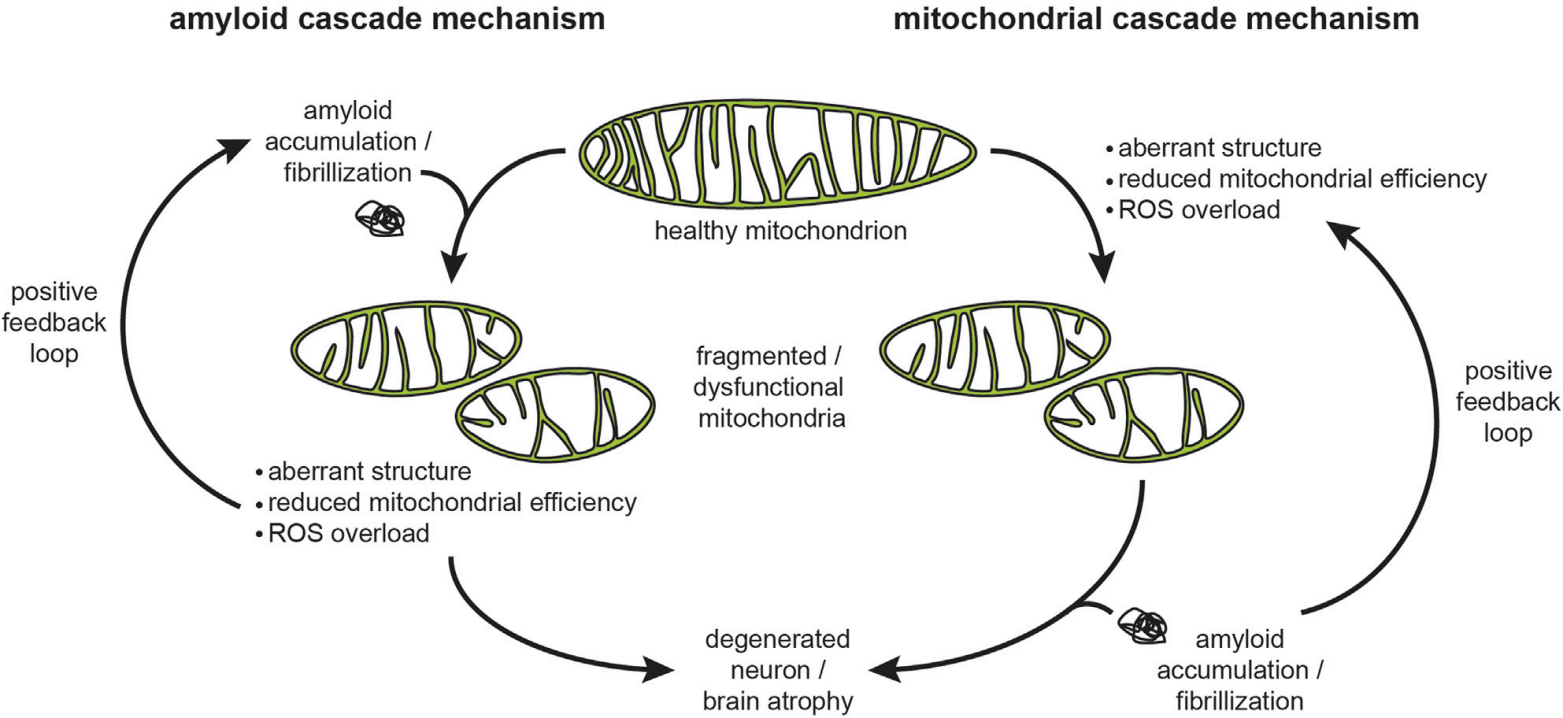
The amyloid and mitochondria cascade hypotheses. These two mechanisms describe the cause-and-effect relationships in the progression of neurodegenerative diseases, proposed originally for AD but also relevant for other proteinopathies. The amyloid cascade hypothesis proposes that amyloid accumulation, fibrillization, and plaque formation trigger the pathogenic events, including mitochondrial damage, that lead to neuronal damage. As a corollary to this hypothesis, the most effective interventions would target the accumulation, clearance, and fibril formation of amyloids. The mitochondria cascade hypothesis proposes that mitochondrial dysfunction is the primary driver of neurodegeneration that leads to cellular damage, including amyloid aggregation. As a corollary to this hypothesis, the most effective interventions would be those aimed toward improving mitochondrial physiology. In both mechanisms, amyloid aggregation and mitochondrial dysfunction can be part of a vicious cycle that accelerates disease progression. Note that the interaction of amyloidogenic proteins with the mitochondrial import machinery likely plays a central role in both mechanisms.

The concept that insoluble fibrils, whether extra- or intra-cellular, are the primary cytotoxic factors that initiate amyloidogenic disease is the core tenet of the “amyloid cascade hypothesis” (Figure 5, left) first proposed by Hardy and Higgins to describe the role of Aβ amyloids in AD pathogenesis (Hardy and Higgins, 1992). Subsequently it was proposed that α-syn fibrils are the primary cytotoxic factors in PD (Stefanis, 2012) and that amyloid fibril-like inclusions of mHtt are the primary cytotoxic factors in HD (Scherzinger et al., 1999). There is considerable controversy, however, on whether amyloid fibrils are the only, or even the principal culprits in pathology. A confounding factor is the heterogeneity of species associated with amyloid formation. Amyloidogenesis involves a hierarchy of structures that starts from the functional, soluble form of a protein or peptide and proceeds to oligomers, nuclei, β-sheets, protofilaments, protofilament bundles, and finally mature fibrils (Serpell, 2000) (Figure 4). Within the fibrils themselves, there are structural polymorphs that can differ depending on whether they are grown *in vitro* or isolated from patients. In fact, increasing evidence suggests distinct structural polymorphs are associated with specific disease subtypes. Therefore, considerable uncertainty surrounds which species represent the culprits in pathogenicity (Carulla et al., 2005; Lansbury and Lashuel, 2006; Haass and Selkoe, 2007; Zraika et al., 2010), with some recent proposals advancing that soluble oligomers (Larson and Lesné, 2012; Arbor et al., 2016; Cline et al., 2018) or even individual proteins/peptides (Hoffner and Djian, 2014; Hillen, 2019) could initiate the phenotypic cascade of amyloidogenic diseases. Because mature fibrils are insoluble and extremely stable, many view soluble oligomeric precursors as the most likely candidates for cytotoxicity. However, studies on amyloid oligomers have been hampered by the low concentrations and transitory nature of these intermediates (Engel, 2009), thereby contributing to a lack of evidence for this model. Finally, it unclear whether cytotoxicity is associated with intracellular or extracellular forms of amyloidogenic proteins (Cuello, 2005; Gurlo et al., 2010; Aston-Mourney et al., 2011).

Much of the current research on the mechanisms of amyloidogenic diseases has shifted focus towards processes preceding mature fibril formation. As a result, it has now been widely demonstrated that mitochondrial dysfunction occurs early in the pathogenesis of AD (Wang et al., 2020b), PD (Malpartida et al., 2021), and HD (Carmo et al., 2018). The “mitochondrial cascade hypothesis”, originally proposed by Swerdlow and colleagues, proposes that it is in fact the progressive decline in mitochondrial function that promotes AD pathology and influences the progression of the disease (Swerdlow et al., 2014) (Figure 5, right). Therefore, exactly how amyloidogenic proteins interact with mitochondria, and specifically how they may engage the mitochondrial protein import machinery, is a key mechanistic question in understanding the pathology of neurodegenerative disorders.

## 4 Neurodegenerative diseases and pathogenic mechanisms of amyloids

Aβ, α-syn, and Htt/mHtt are among the approximately 50 amyloidogenic proteins associated with human diseases (Chiti and Dobson, 2017). In this section, we review the roles of these proteins in AD, PD, and HD, respectively, with a special emphasis on their interactions with mitochondria.

### 4.1 Alzheimer’s disease and the roles of APP and Aβ

**FIGURE 6:**
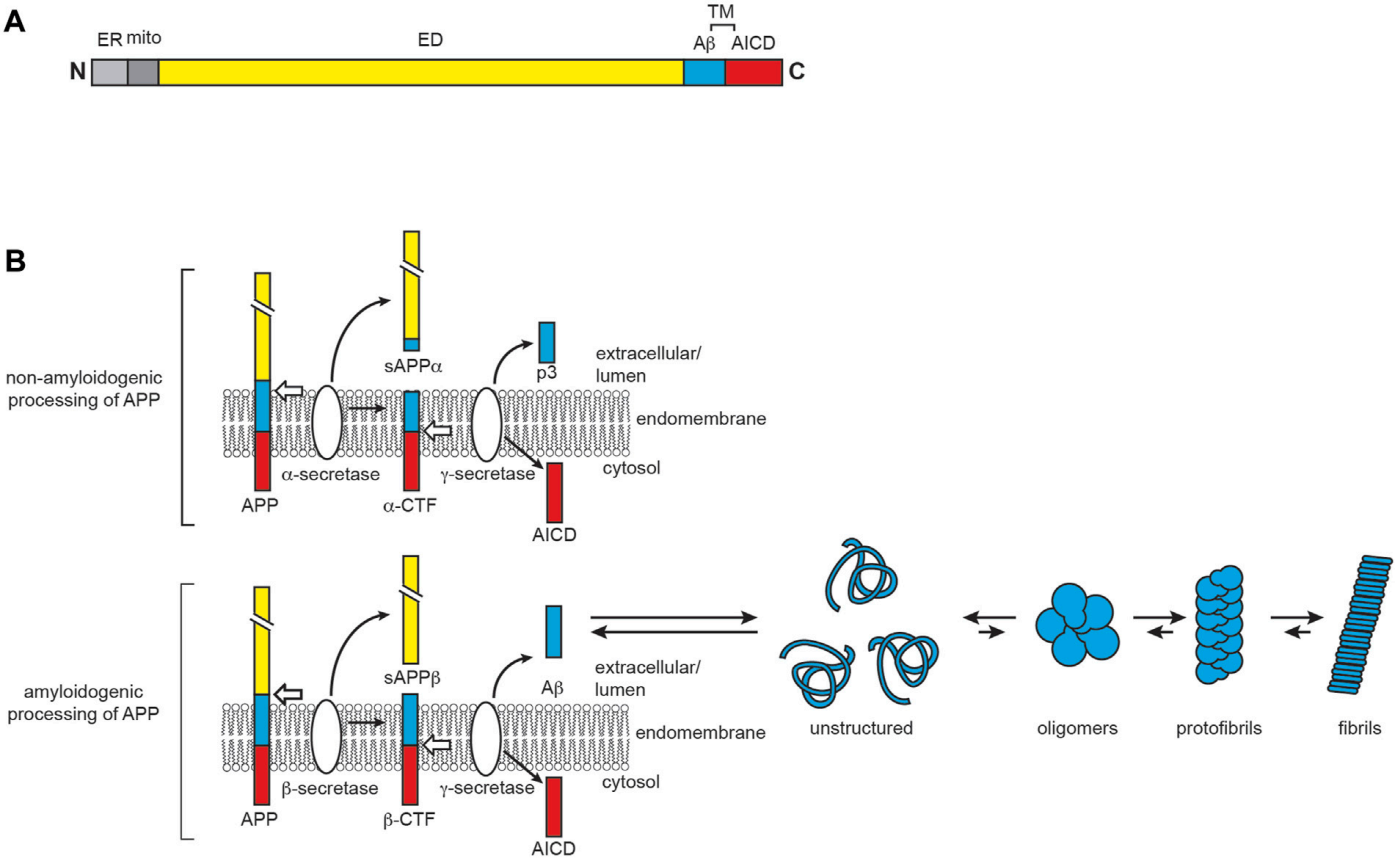
AD and the role of APP and Aβ. **(A)** APP domain organization. Full-length APP contains an extracellular domain (ED, yellow), a transmembrane region (TM), and the APP intracellular domain (AICD, red). The proteolysis product corresponding to Aβ is shown in cyan. The N-terminal ER- and mitochondria-targeting sequences are shown in light and dark gray, respectively. **(B)** APP processing and Aβ aggregation. APP has two main proteolytic fates. In the non-amyloidogenic pathway (top), APP is first cleaved by α-secretase to yield soluble APPα (sAPPα) and the α-C-terminal fragment (α-CTF), the latter of which is then cleaved by γ-secretase to produce AICD and the non-amyloidogenic extracellular peptide p3. In the amyloidogenic pathway (bottom), β-secretase proteolyzes full-length APP to yield soluble APPβ (sAPPβ) and the β-C-terminal fragment (β-CTF), the latter of which is then cleaved by γ-secretase to produce AICD and the amyloid beta peptide Aβ. The Aβ peptide, particularly Aβ_42_, is disordered in solution and prone to aggregation and fibrillization.

AD is the leading cause of senile dementia, affecting several regions of the brain involved in memory and cognition, including the hippocampus, the neocortex, and the basal forebrain (Auld et al., 2002). While the exact etiology of AD is unknown, it is biologically characterized by the aggregation of two misfolded proteins: Aβ, the primary component of extracellular plaques, and hyperphosphorylated variants of the microtubule-associated protein tau, which form intracellular inclusions known as neurofibrillary tangles (NFTs) (Knopman et al., 2021). Aβ is produced through sequential cleavage of APP, a membrane glycoprotein with isoforms ranging from 100 to 140 kDa that predominantly reside at neuronal synapses (Zhou et al., 2011; Hoe et al., 2012) (Figure 6A). APP plays a vital role in neural development and synaptic plasticity, as it is implicated as a receptor involved in kinesin 1 cargo recognition (Lazarov et al., 2005), the Wnt signaling pathway (Liu T. et al., 2021) and other functions including cell adhesion, synaptogenesis (Baumkötter et al., 2014), and iron export (Duce et al., 2010). As a type I integral membrane protein, APP has a large ectodomain at its N-terminus, a single α-helical transmembrane segment, and a small C-terminal intracellular domain. APP belongs to a highly conserved superfamily of genes (Coulson et al., 2000; Jacobsen and Iverfeldt, 2009). In mammals, alternative splicing generates eight APP isoforms, the three most common being APP_695_, APP_751_, and APP_770_, among which APP_695_ is most highly expressed in neurons (Sandbrink et al., 1996; Belyaev et al., 2010). Like other plasma membrane proteins, the life cycle of APP following synthesis consists of membrane trafficking via the secretory pathway and degradation and recycling by the endocytic system (Lin et al., 2021). The Aβ peptides that form amyloid plaques in AD are derived from the processing of APP through sequential actions of β- and γ-secretases (Selkoe and Hardy, 2016; Zhao et al., 2020) (Figure 6B). The generated Aβ peptides range in size, with the 42-residue Aβ_42_ being much more aggregation-prone than the more abundant 40-residue Aβ_40_ (Seubert et al., 1992; Haass and Selkoe, 2007; Chow et al., 2010). Monomeric Aβ_40_ and Aβ_42_ are intrinsically disordered in solution (Roche et al., 2016), with Aβ_42_ having a transient population of β-sheet structure (Kakeshpour et al., 2021).

The neuropathology of AD has historically been described by the amyloid cascade hypothesis, whereby Aβ deposition is the causative event (Hardy and Higgins, 1992). In support of this hypothesis, early-onset familial AD (FAD) is caused by autosomal dominant inheritance mutations in the genes encoding APP or APP processing enzymes (Bekris et al., 2010; Ricciarelli and Fedele, 2017). However, the vast majority of cases are sporadic, late onset AD (LOAD) occurring without mutations in the genes encoding APP or APP processing enzymes (Bekris et al., 2010). While the correlation between Aβ and AD remains strongly supported (Herrup, 2015), intracellular Aβ in the form of soluble monomers and oligomers are now considered to be the primary toxic species rather than insoluble, fibrillar assemblies of Aβ that form extracellular plaques (Goure et al., 2014; Gallego Villarejo et al., 2022). Despite this, soluble Aβ species precede the formation of plaques, which are found in patient brains prior to the onset of clinical symptoms, and it has been shown that Aβ alone is not sufficient to induce disease pathogenesis (Herrup, 2015). This suggests a more complex pathological mechanism at play that led to the development of a variety of alternative hypotheses surrounding AD etiology**.**

One prevailing proposal is the ApoE cascade hypothesis (Martens et al., 2022), based on the genetic association of LOAD with the apolipoprotein ε4 allele (APOE4) (Corder et al., 1993). APOE4 carriers have a vastly increased risk of developing LOAD and a reduced age of onset compared to carriers of the APOE3 and APOE2 isoforms (Corder et al., 1993; Sando et al., 2008). ApoE is a 34 kDa lipid-binding protein that primarily functions in lipid transport and metabolism (Mahley, 2016), with differences among the ApoE2, E3, and E4 isoforms confined to two sites (residues 112 and 158) (Najm et al., 2019; Martens et al., 2022). The primary functional difference in ApoE4 is its binding preference for very-low density lipoprotein (VLDL) over high-density lipoprotein (HDL), whereas the ApoE2 and ApoE3 isoforms preferentially bind HDL (Raffai et al., 2001; Belloy et al., 2019). While ApoE contributes to Aβ synthesis, accumulation, and clearance in an isoform-dependent manner, there is conflicting evidence on the isoform-specific roles of ApoE in Aβ pathology (Koistinaho et al., 2004; Castellano et al., 2011; Huang and Mahley, 2014; Najm et al., 2019). The ApoE cascade hypothesis does not include Aβ as a contributing factor in disease pathogenesis, stating that the biophysical and structural properties dependent of the ApoE isoform initiate a cascade of events driving AD and aging-related pathogenic condition (Martens et al., 2022). ApoE4 has been shown to alter lipid homeostasis and metabolism, leading to changes in lipid droplet formation, cholesterol turnover, and the PC/PE ratio (Farmer et al., 2019; Lazar et al., 2022; Yang et al., 2023). Consistent with MAM alterations, several lines of work support the involvement of ApoE in mitochondrial dysfunction as a contributing component to disease pathogenesis, including recent findings suggesting mitochondrial dysfunction influences ApoE expression and secretion (Dose et al., 2016; Martens et al., 2022; Wynne et al., 2023).

AD is associated with pronounced defects in mitochondrial function (Cenini and Voos, 2019; Wang et al., 2020c). These include alterations in mitochondrial biogenesis and morphology, and decreases in mitochondrial number (Hirai et al., 2001; Qin et al., 2009; Oka et al., 2016; Brustovetsky et al., 2023); compromised energy metabolism, including glucose hypometabolism (Kumar et al., 2022) and deterioration of the TCA and OXPHOS systems (particularly CIV) (Bubber et al., 2005; Liang et al., 2008; Zhang et al., 2015; Mastroeni et al., 2017; Sorrentino et al., 2017; Adav et al., 2019; Ryu et al., 2021); oxidative stress (Misrani et al., 2021); effects on mitochondrial dynamics, most notably a general increase in fragmentation (Wang et al., 2008a; Wang et al., 2009); impairment of mitochondrial trafficking (Calkins et al., 2011); and altered ER-mitochondria apposition with associated cellular Ca^2+^ dyshomeostasis (Leal et al., 2020; Fernandes et al., 2021; Li et al., 2023). While these effects could be associated with the interaction of APP/Aβ with mitochondria (see below), other AD-related factors could also be involved.

### 4.2 Parkinson’s disease and the role of α-syn

**FIGURE 7:**
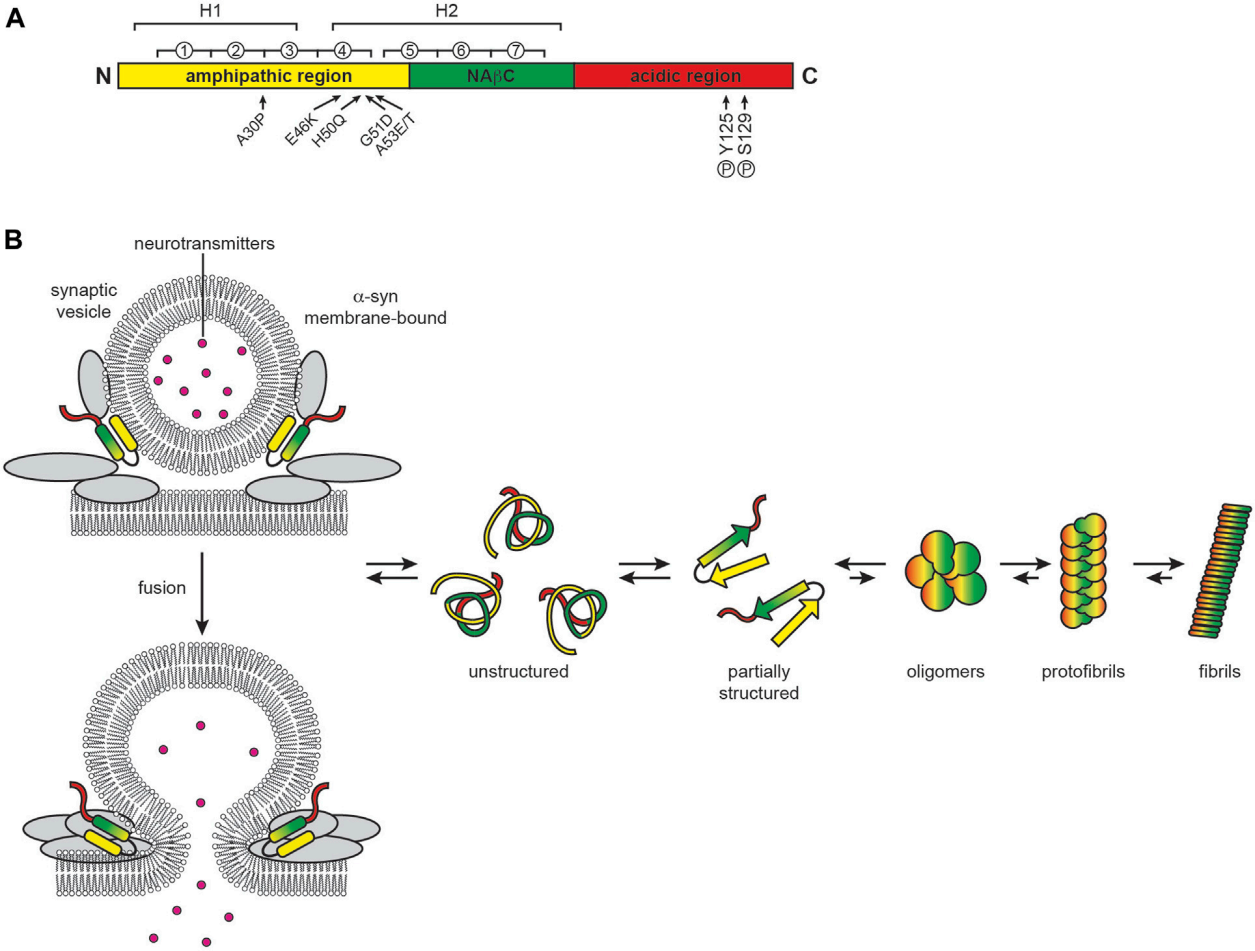
PD and the role of α-syn. **(A)** α-syn domain organization. α-syn contains an N-terminal amphipathic region involved in membrane interactions (yellow, residues 1–60), a central non-amyloid β component (NAβC) responsible for protein fibrillization (green, residues 61–95) and a highly acidic C-terminal region (red, residues 96–140). Circled numbers indicate the seven 11-residue repeats containing the KTKEGV motif; H1 and H2 indicate the regions of helical propensity that form two helices in the micelle-bound state or can form an elongated helix when bound to lower-curvature membranes. Arrows indicate sites of point mutations and phosphorylation, as indicated, that enhance α-syn misfolding and aggregation **(B)** α-syn function and aggregation. When membrane bound, α-syn folds into a single elongated helix or forms a two-helix structure. In solution, α-syn is largely disordered and prone to aggregation and fibrillization.

PD involves the selective degeneration of nigrostriatal dopaminergic neurons, manifesting as progressive effects on movement that include rigidity, resting tremor, and bradykinesia (Zhai et al., 2018). The main histological features of PD are cytoplasmic inclusions called Lewy bodies (LBs) and neuritic inclusions called Lewy neurites (LNs) whose core component is α-syn (Baba et al., 1998; Spillantini et al., 1998) but also contain vesicular structures, membranes and fragmented organelles (Shahmoradian et al., 2019; Ericsson et al., 2021). These α-syn aggregates are characteristic of a general group of neurodegenerative disorders termed synucleinopathies. α-syn is a 140 residue protein encoded by the synuclein alpha (*SNCA*) gene that contains three domains: (i) an N-terminal amphipathic region, (ii) a highly hydrophobic region called the non-amyloid-β component (NAβC), and (iii) a C-terminal acidic region (Bisi et al., 2021) (Figure 7A). Being enriched in the axon terminals of presynaptic neurons, α-syn is a membrane-interactive protein that plays a role in synaptic function, including vesicle trafficking and exocytosis by assembly of SNARE complexes, synaptic membrane remodeling, and maintenance of neurotransmitter vesicle pools (Bendor et al., 2013; Burre, 2015). α-syn also localizes to the nucleus, where it regulates gene expression (Somayaji et al., 2021).

Monomeric α-syn is intrinsically disordered in solution, dynamically interconverting among many different conformations that differ with respect to short- and long-range electrostatic interactions and transient secondary structures (Weinreb et al., 1996). The N-terminus of α-syn in solution has helical propensity with a low fractional population assuming α-helix conformation; however, when bound to membranes, it adopts a stable helical structure that is either a continuous helix or two separate helices (Davidson et al., 1998; Bussell and Eliezer, 2003; Chandra et al., 2003; Ulmer et al., 2005; Croke et al., 2008; Georgieva et al., 2008; Jao et al., 2008; Stockl et al., 2008; Cho et al., 2009; Bodner et al., 2010; Rao et al., 2010; Croke et al., 2011; Ullman et al., 2011; Adao et al., 2020; Musteikyte et al., 2021)**.** Membrane binding is facilitated by seven imperfect repeats of the 11-residue pattern xKTKEGVxxxx that extend from the amphipathic domain into the NAβC domain, a motif also found in apolipoproteins A2 (Bussell and Eliezer, 2003). This membrane-interactive region imparts classic amphitropic behavior to α-syn, which consequently promotes its interaction with curved bilayers, particularly with those enriched in negatively charged phospholipids (Jo et al., 2002) and cholesterol (Fantini et al., 2011). This membrane-binding activity is critical for its function in synaptic vesicle interaction (Fortin et al., 2005; Sarchione et al., 2021). The hydrophobic NAβC domain nucleates α-syn aggregation, forming the canonical β-structure of amyloid fibrils along with parts of the N-terminal domain (Rodriguez et al., 2015). The fraction of α-syn that natively exists in neurons in a membrane-bound state, as an unfolded monomer (Fauvet et al., 2012; Theillet et al., 2016), or as aggregation-resistant folded tetramers (Bartels et al., 2011; Wang et al., 2011) remains an open question.

Familial autosomal dominant PD is linked to defects in several genes, most of which have a connection to mitochondrial physiology (Cieri et al., 2017; Billingsley et al., 2018). Among them, the six known heritable mutations in the *SNCA* gene (A30P, E46K, H50Q, G51D, and A53E/T) all localize to the amphipathic N-terminal region of α-syn and promote aggregation (Flagmeier et al., 2016). α-syn aggregation can also be potentiated by increases in its copy number (Srinivasan et al., 2021). However, PD is primarily an idiopathic disease in which aging-related increases in α-syn aggregation and/or certain post-translational modifications of α-syn are commonly involved (Anderson et al., 2006; Manzanza et al., 2021; Roshanbin et al., 2021). The balance between α-syn function and pathogenicity is depicted in Figure 7B.

Mitochondrial dysfunction plays a key role in the pathogenesis of PD (Vicario et al., 2018). The enhanced expression of α-syn or particular mutants of α-syn known to promote PD cause the disruption of mitochondrial dynamics (Xie and Chung, 2012; Thorne and Tumbarello, 2022) and promote fragmentation (Kamp et al., 2010; Nakamura et al., 2011; Gui et al., 2012; Pozo Devoto et al., 2017), impair mitochondrial trafficking (Pozo Devoto et al., 2017) and autophagic clearance (Gao et al., 2017), impact mitochondrial energetics (Banerjee et al., 2010), and alter Ca^2+^ flux by the enhancement (Cali et al., 2012) or disruption (Guardia-Laguarta et al., 2014; Guardia-Laguarta et al., 2015; Paillusson et al., 2017) of specific ER-mitochondria tethering sites. One of the most prominent effects of PD is the impairment of respiratory Complex I associated with reduced energetic output and an overproduction of ROS (Devi et al., 2008; Marella et al., 2009). This multitude of effects is in line with the many mitochondrial subcompartments α-syn has been found to target, including the MOM (Li et al., 2007; Cole et al., 2008; McFarland et al., 2008; Nakamura et al., 2008; Zhang et al., 2008; Kamp et al., 2010; Nakamura et al., 2011; Di Maio et al., 2016a; Pozo Devoto et al., 2017), the MIM/IMS (McFarland et al., 2008; Nakamura et al., 2011; Zhu et al., 2011; Lu et al., 2013; Robotta et al., 2014; Amorim et al., 2017), the matrix (McFarland et al., 2008; Ludtmann et al., 2016; Ludtmann et al., 2018) and the MAM (Cali et al., 2012; Guardia-Laguarta et al., 2014; Paillusson et al., 2017). Importantly, this extensive interaction of α-syn with mitochondria is mediated in part through lipid bilayer interactions, particularly with regions enriched with the mitochondrial lipid cardiolipin (Ramakrishnan et al., 2003; Cole et al., 2008; Nakamura et al., 2008; Grey et al., 2011; Zigoneanu et al., 2012; Robotta et al., 2014; Ryan et al., 2018) that provides the negative surface, high curvature, and acyl packing defects necessary to promote α-syn binding (Sharon et al., 2003; Nuscher et al., 2004; Middleton and Rhoades, 2010; Pfefferkorn et al., 2012; Ouberai et al., 2013; Gilmozzi et al., 2020).

### 4.3 Huntington’s disease and the role of mHtt

**FIGURE 8:**
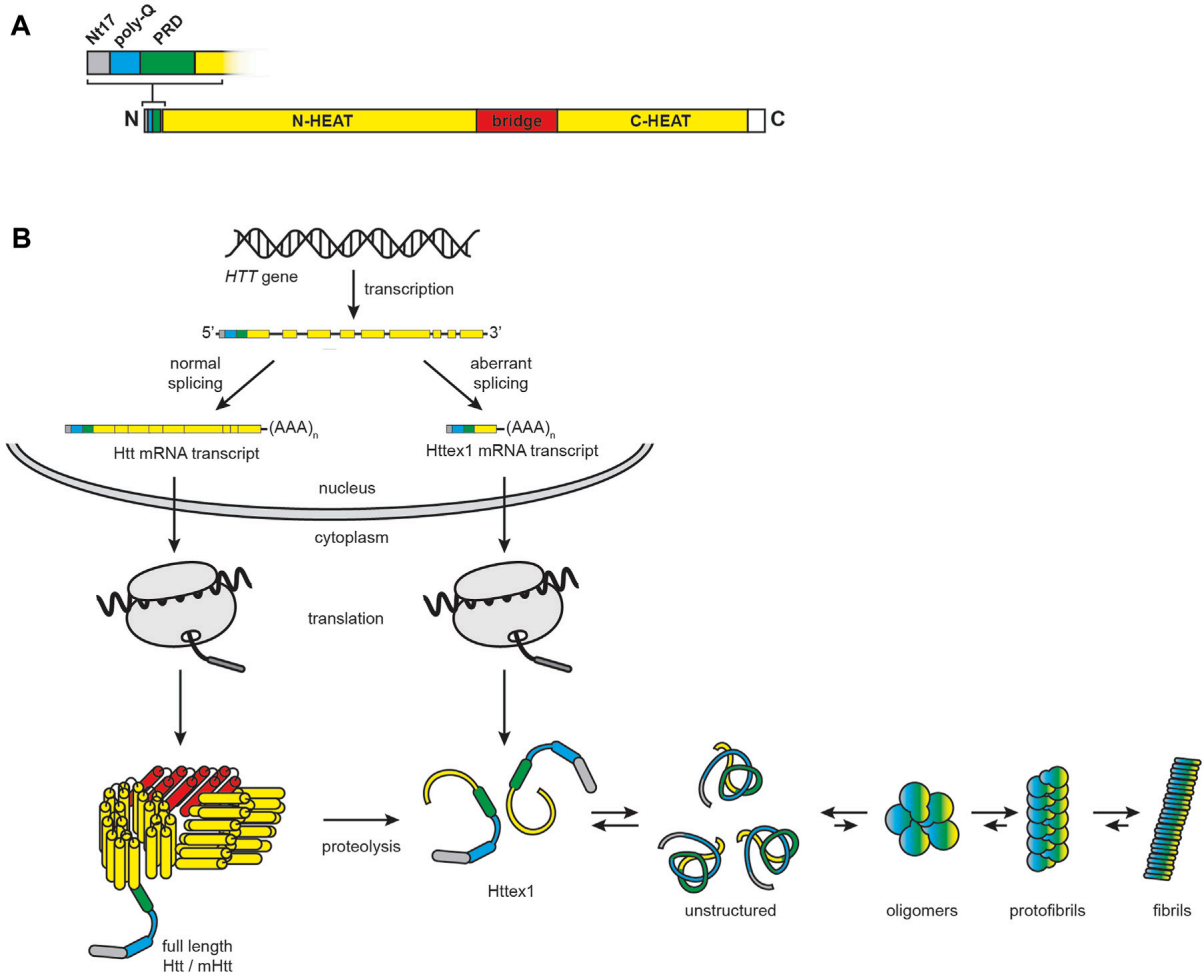
HD and the role of mHtt. **(A)** Htt domain organization. Full-length huntingtin is a 3144-residue protein that consists of N-terminal (residues 91–1683) and C-terminal (residues 2092–3057) α-helical solenoid domains containing multiple HEAT (huntingtin, elongation factor 3, protein phosphatase 2A, lipid kinase TOR) repeats (yellow) connected by a helical linker region (red, residues 1684–2091). The full-length protein also features an N-terminal region consisting of the Nt17 sequence (grey, residues 1–17), the poly-Q tract, which is expanded in HD (cyan, residues 18–40), and a proline-rich domain (green, residues 41–90). **(B)** Htt biogenesis, processing, and misfolding. In the nucleus, normal splicing of the *HTT* gene results in a full-length transcript, whereas alternative splicing and premature polyadenylation results in a transcript encoding Httex1. Following translation in the cytosol, full-length Htt is subject to extensive proteolysis, which also contributes to the pool of Httex1 protein. Httex1 is unstructured in solution and prone to aggregation and fibrillization.

HD is associated with neurodegeneration of the basal ganglion, with preferential deterioration of striatal medium spiny GABAergic neurons, that clinically presents as progressive loss of motor control and cognition (Morigaki and Goto, 2017). This monogenic disease is caused by heritable alterations in Htt (Jurcau and Jurcau, 2022). Structurally, Htt is a 348 kDa protein that contains two domains of HEAT tandem repeats that form α-solenoid structures connected by a bridge domain, and an N-terminal region with a tripartite organization that contains a highly conserved sequence of 17 N-terminal amino acids (Nt17), a stretch of glutamine residues (poly-Q), and a proline-rich domain (PRD) (Saudou and Humbert, 2016; Guo et al., 2018) (Figure 8A). Htt is conformationally dynamic, with an extensive list of interaction partners likely owing to the protein-interaction functions of its HEAT repeats (Shirasaki et al., 2012). As such, it serves as a multivalent molecular scaffold, a function that likely supports its many interactions in synapses including axonal transport, vesicle recycling, autophagy, transcriptional regulation, and endocytosis (Barron et al., 2021).

HD is an autosomal dominant disorder caused by an extension of the poly-Q tracts resulting from polymorphic CAG trinucleotide repeat expansion in exon 1 of the *HTT* gene (MacDonald et al., 1993). HD is part of a larger family of neurodegenerative diseases associated with polyglutamine expansion of proteins (Lieberman et al., 2019). Whereas Htt in healthy individuals contains fewer than 36 CAG repeats, the pathogenically expanded poly-Q region can contain between 42 and 250 CAG repeats, with disease severity and earlier age of onset directly related to the extent of expansion (Rubinsztein et al., 1996; Penney et al., 1997). Because Htt is ubiquitously expressed, HD affects not only the brain but also peripheral tissues with high metabolic activity (Chuang and Demontis, 2021). Poly-Q expansion causes the resulting mHtt protein to have altered folding (Vijayvargia et al., 2016) and interactions (Ratovitski et al., 2012; Greco et al., 2022). This makes mHtt prone to aggregation, forming inclusion bodies in the cytoplasm and nucleus (Davies et al., 1997) with diverse aggregate structures (Tanaka et al., 2001; Poirier et al., 2002) and a complex composition that includes lipids, proteins, and membrane-bound organelles (Kegel-Gleason, 2013; Riguet et al., 2021). Notably, the primary cytotoxic, aggregation-prone species in HD may not be full-length mHtt, but rather heterogeneous populations of N-terminal fragments of mHtt exon 1 (Httex1) (Mangiarini et al., 1996; Cooper et al., 1998; Hackam et al., 1998; Martindale et al., 1998; Hoffner et al., 2005; Barbaro et al., 2015) that can arise by cleavage of the mature protein by caspase, calpain, or other proteases (Goldberg et al., 1996; Wellington et al., 1998; Kim et al., 2001; Gafni and Ellerby, 2002; Lunkes et al., 2002; Graham et al., 2006; Schilling et al., 2007; Landles et al., 2010; Tebbenkamp et al., 2012; Martin et al., 2019), or by aberrant splicing and premature polyadenylation of pathogenic *HTT* exon 1 with expanded CAG repeats (*HTT*ex1 transcripts) (Sathasivam et al., 2013; Neueder et al., 2017; Neueder et al., 2018) (Figure 8B).

Mitochondrial dysfunction plays a central role in HD pathogenesis (Bossy-Wetzel et al., 2008; Carmo et al., 2018; Jurcau and Jurcau, 2023). HD is associated with reduced mitochondrial biogenesis and quality control (Steffan et al., 2000; Cui et al., 2006; Weydt et al., 2006; Jin and Johnson, 2010; Wong and Holzbaur, 2014; Khalil et al., 2015; Guo et al., 2016; Dubois et al., 2021; Sonsky et al., 2021), mtDNA heteroplasmy (Wang et al., 2021), defective energy metabolism and OXPHOS activity (Sawa et al., 1999; Klivenyi et al., 2004; Seong et al., 2005; Benchoua et al., 2006; Pandey et al., 2008; Mochel et al., 2012; Silva et al., 2013; Naia et al., 2015; Burtscher et al., 2020), altered mitochondrial Ca^2+^ handling and sensitivity to the mitochondrial permeability transition pore (mPTP) (Panov et al., 2002; Choo et al., 2004; Milakovic et al., 2006; Giacomello et al., 2013), increased mitochondrial oxidative stress (Sorolla et al., 2008; Chae et al., 2012; Ribeiro et al., 2012; Ribeiro et al., 2014; Chen et al., 2017; Moretti et al., 2021; Lopes et al., 2022; Egidio et al., 2023), defective mitochondrial dynamics and hyper-fission (Costa et al., 2010; Song et al., 2011; Shirendeb et al., 2012; Manczak and Reddy, 2015; Cherubini et al., 2020), defective mitochondrial trafficking (Trushina et al., 2004; Chang et al., 2006; Orr et al., 2008; Shirendeb et al., 2012; Berth and Lloyd, 2023), and altered cytochrome *c* release with apoptosis (Chen et al., 2000; Kiechle et al., 2002; Zhang et al., 2006; Wang et al., 2008b). Although many of these effects are related to spurious interactions of mHtt with cytosolic proteins, there are several reports that full-length and N-terminal fragments of Htt/mHtt interact directly with mitochondria (Panov et al., 2002; Choo et al., 2004; Petrasch-Parwez et al., 2007; Orr et al., 2008; Song et al., 2011; Guo et al., 2016). Some of these studies specifically implicate interactions with the MOM (Hamilton et al., 2020) and some show interactions with internal mitochondrial compartments (Yano et al., 2014; Yablonska et al., 2019). In general, accumulation of N-terminal mHtt fragments with mitochondria increases with age (Orr et al., 2008). Potential interactions between Htt/mHtt and the mitochondrial import machinery are discussed below.

## 5 Interactions of amyloidogenic proteins with the mitochondrial protein import machinery

### 5.1 Noncanonical and multi-specific targeting signals

**FIGURE 9:**
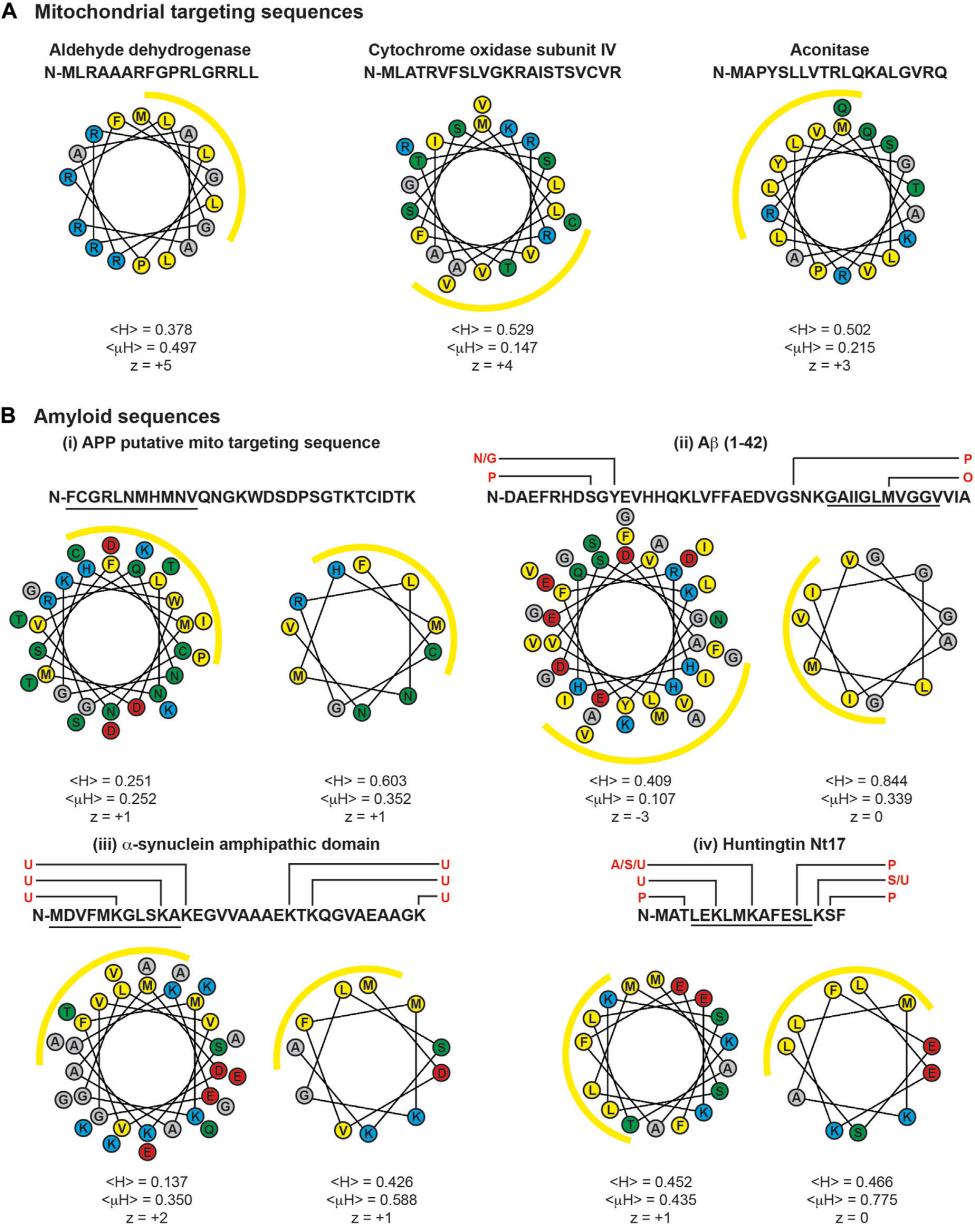
MTSs and cryptic targeting sequences of amyloids. Sequences and helical wheel diagrams of MTSs and select regions of amyloidogenic proteins are depicted. Helical projections and parameters were determined using the HeliQuest server (Gautier et al., 2008). Mean hydrophobicity is denoted as <H>, hydrophobic moment as <μH>, and net charge as z. For all projections, the N-terminal residue is oriented due North (0^o^), and yellow arcs indicate the hydrophobic face of the amphipathic helices determined by <μH> values. **(A)** Example MTSs. Human variants of select TIM23 substrates aldehyde dehydrogenase, subunit IV of Cytochrome *c* oxidase, and aconitase are shown. **(B)** Cryptic targeting signals of amyloidogenic proteins. These include the putative MTS of: (i) APP, (ii) Aβ (1-42), (iii) α-syn (1-32), and (iv) huntingtin (1-17). Helical projections to the left show the entire sequence. Helical projections to the right show the 11-residue (∼3 helical turn) section with the strongest amphipathic character, indicated by the underlined residues. Known sites of post-translational modification are indicated above the primary sequences in red (A, acetylation; G, glycosylation; N, nitration; O, oxidation; P, phosphorylation; S, SUMOylation; U, ubiquitinylation). Single letter amino acid codes colored by side chain functionality: yellow, hydrophobic; red, acidic; cyan, basic; green, polar uncharged.

Most proteins that are targeted to cellular compartments other than their site of ribosomal synthesis contain unambiguous targeting sequences that faithfully direct them to a specific location. For example, as described above, the MTSs of TIM23 substrates form an N-terminal amphipathic α-helix that serves as the primary recognition element of the TIM23 import machinery. Three examples of MTSs shown in Figure 9A illustrate their amphipathic character: they have an appreciable number of nonpolar residues quantified as the mean hydrophobicity (<H>) (Fauchere and Pliska, 1983), a net charge (z) that reflects a preponderance of basic residues, and an asymmetric distribution of nonpolar and basic residues quantified by the hydrophobic moment (<μH>) (Eisenberg et al., 1982). Yet it is also notable that MTSs vary significantly with respect to sequence, length, and extent of amphipathicity. Thus, while canonical MTSs encompass a range of physicochemical properties, they are sufficiently well-defined enough to be recognized by a single import pathway.

For some proteins, however, the relationship between the targeting sequence and the organellar import pathway is not straightforward and some targeting sequences can direct passenger proteins to multiple cellular compartments by non-mutually exclusive mechanisms (Karniely and Pines, 2005). Indeed, targeting fidelity requires multiple levels of regulation and precise recognition because mitochondrial MTSs, ER signal peptides, chloroplast transit peptides, and even some peroxisomal targeting signals share similarity in that they all form N-terminal amphipathic α-helices with a hydrophobic face and a basic (or polar) face (Kunze and Berger, 2015).

Some proteins contain an ambiguous (or cryptic) targeting sequence that can be recognized by multiple import machineries (Lu et al., 2004; Ventura et al., 2004; Chatre et al., 2009). Other proteins contain well-defined targeting signals whose accessibility or targeting efficiency can be modified by folding, protein binding, or post-translational modifications (Karniely and Pines, 2005). Still other proteins contain multiple targeting signals that direct them to different compartments (Petrova et al., 2004; Pino et al., 2007). For example, some cytochromes P450 (CYPs) expressed in hepatocytes contain chimeric signals with amino-terminal ER-targeting sequences in tandem with a mitochondrial-targeting sequence (Addya et al., 1997; Anandatheerthavarada et al., 1999; Robin et al., 2001; Robin et al., 2002). The bipartite targeting sequence of these cytochromes contain a cryptic mitochondria localization signal that remains idle until activation. In the case of CYP2E1, the mitochondrial targeting signal is activated by cyclic-AMP-dependent phosphorylation of a Ser residue in the cryptic sequence (Robin et al., 2002). Moreover, an inducible endoprotease has been identified that activates mitochondrial import by cleaving bimodal targeting signals to expose the cryptic mitochondrial targeting signal (Boopathi et al., 2008). It is unclear whether such cleavage of chimeric signals is a general requirement for mitochondrial targeting.

By allowing greater diversity of potential cellular locations, noncanonical targeting imparts flexibility to the functional range of individual proteins that can be subject to regulation by the cell in response to different physiological demands. However, cryptic targeting sequences can also cause the pathogenic accumulation of proteins in organelles. In the following sections, we review the cryptic mitochondria targeting sequences present in Aβ/APP, α-syn, and Htt/mHtt that may facilitate their interactions with the mitochondrial import machinery.

### 5.2 Interactions of Aβ and APP with the mitochondrial import machinery

**FIGURE 10:**
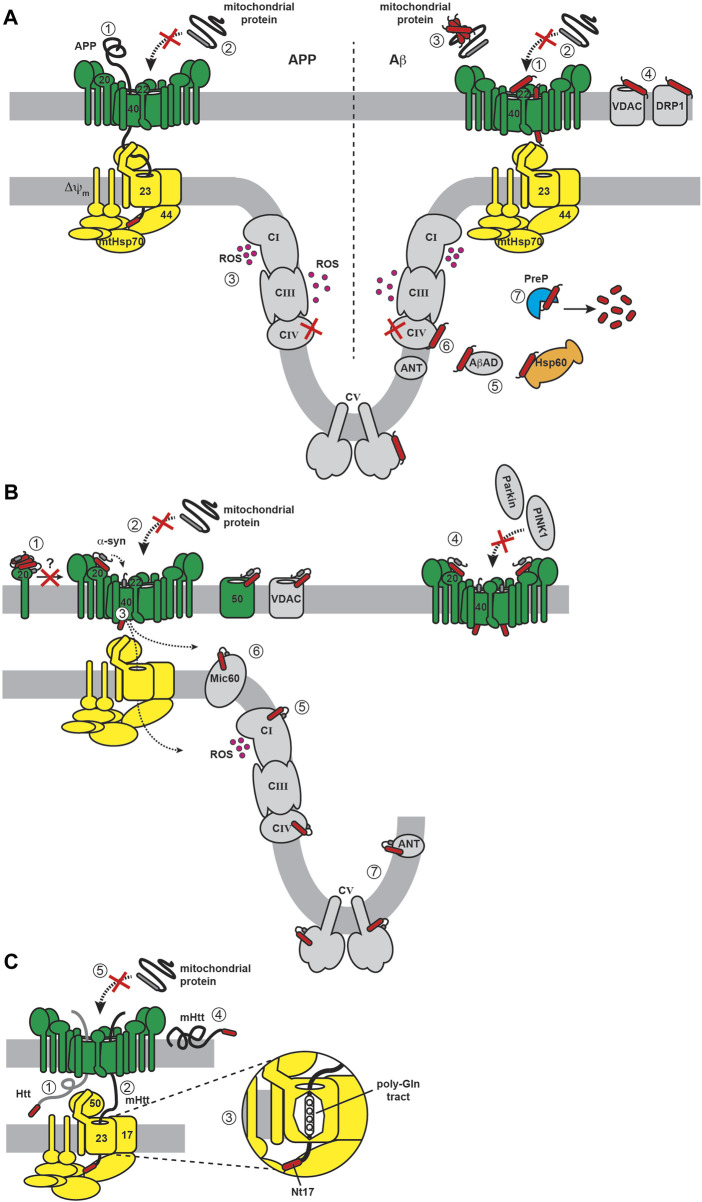
Interactions of amyloidogenic proteins with the TIM23 complex. Models show import complex color scheme used in Figure 2 (TOM/SAM complex, green; TIM23 complex, yellow) with other proteins depicted in grey. Subunits known to interact with amyloids are explicitly indicated by their names. Putative cryptic targeting signals of Aβ/APP, α-syn, and Htt/mHtt are shown in red. [**(A)**, left] APP. (1) APP forms a two membrane-spanning intermediate, engaging both TOM and TIM23 complexes in a Δψ_m_-dependent manner, with complete translocation blocked by the tight folding of the C-terminal AICD domain. (2) Stalled APP intermediates block the import of native mitochondrial precursor proteins. (A, right) Aβ. (1) Aβ interacts with the TOM complex (subunits Tom22 and Tom40), which (2) disrupts the import of native mitochondrial precursors. (3) Aβ forms co-aggregates with mitochondrial precursors in the cytosol. Aβ interacts with proteins of the (4) MOM, (5) matrix, and (6) MIM, most notably CIV and CV. (7) Aβ is degraded by the matrix PreP protease. A general feature of APP/Aβ stress is dysfunctional CIV and excess ROS production by the OXPHOS machinery. **(B)** α-syn. (1) Multimers of α-syn interact with free Tom20, blocking its assembly with the TOM complex. The engagement of α-syn with TOM complex receptors and channel may (2) hinder import of native mitochondrial proteins, (3) reduce Tom40 expression, and/or (4) disrupt PINK1/Parkin-mediated mitophagy; (5) Interaction of α-syn with CI or CIV may disrupt ETC activity and cause ROS overproduction; (6) Interaction of α-syn with MICOS subunit Mic60 may affect cristae morphogenesis; (7) Interaction of α-syn with ATP synthase (CV) or the adenine nucleotide translocase (ANT) may disrupt adenine nucleotide flux and/or ATP production. **(C)** Htt/mHtt. (1) Htt may engage the TOM machinery with a tendency to accumulate in the IMS. (2) mHtt engages the TIM23 complex, (3) possibly facilitated by the unstructured poly-Q segment C-terminal to Nt17, which provides a flexible linker for Nt17 to engage TIM23 subunits. (4) mHtt may alternatively accumulate on the cytosolic side of the MOM. (5) Blockage of the import machinery by Htt/mHtt hinders import of mitochondrial precursors.

Several lines of evidence indicate that full-length APP engages the TIM23 import pathway. The canonical biogenesis route of APP entails targeting to the ER via the SRP/Sec61 pathway, translocation across the ER membrane attendant with glycosylation of the ectodomain, integration as a type I membrane protein, and trafficking by the secretory, endocytic and recycling routes (Muresan and Ladescu Muresan, 2015). APP contains a classic N-terminal Sec-targeting signal with a predicted Signal Peptidase cleavage site (AxA) between positions 17 and 18 (Auclair et al., 2012; Almagro Armenteros et al., 2019). Following this ER-targeting signal is a potential cryptic MTS spanning residues 37 to 67, segments of which could form an amphipathic helix (Figure 9Bi). Thus, together they form a chimeric ER-mitochondria targeting signal (Devi and Anandatheerthavarada, 2010). Indeed, it has been shown using human cortical neuronal cells that a non-glycosylated form of APP_695_ can stall in the mitochondrial import pathway with a predicted N^mito^/C^cyto^ topology. This stalled intermediate made crosslinking-detected interactions with Tom40, Tim23, and Tim44, in a manner that required the Δψ_m_ and the positive residues Arg40, His44, and Lys51, all three of which reside on a common face of the putative MTS (Anandatheerthavarada et al., 2003) (Figure 9Bi). Similar translocation intermediates of APP were observed in mitochondria of transgenic AD mouse models (Anandatheerthavarada et al., 2003) and in postmortem samples of AD brains (Devi et al., 2006), the latter showing stable association of APP with ∼480 kDa complexes containing the TOM machinery and ∼620 kDa complexes containing the TIM23 machinery. The stalling of APP in the import machinery appeared to be due to the tight folding of the APP acidic domain (residues 220–290) blocking transport along the TOM complex, as deletion of this domain facilitated complete translocation (Anandatheerthavarada et al., 2003). This observation is consistent with the formation of stalled translocation intermediates observed with native mitochondria-targeted substrates with tightly folded (cofactor-bound) C-terminal domains and fusion constructs with MTSs fused to cofactor-stabilized domains like DHFR (Schneider, 2018). In agreement with the known effects of AD on mitochondria function, APP translocation intermediates were shown to interfere with the import of native mitochondrial proteins (Devi et al., 2006). The extent to which mitochondrial targeting of APP may be facilitated by endoprotease cleavage of the ER targeting signal to expose the cryptic MTS (Boopathi et al., 2008) is an open question. Figure 10A (*left*) summarizes the interactions of APP with mitochondria.

The Aβ peptide has also been widely reported to accumulate in mitochondria through the import machinery. The presence of Aβ within mitochondria is well documented by its direct interactions with many mitochondrial proteins, including MOM proteins VDAC (Manczak and Reddy, 2012) and Drp1 (Manczak et al., 2011), matrix proteins Hsp60 (Caspersen et al., 2005), Aβ-binding alcohol dehydrogenase (ABAD) (Lustbader et al., 2004) and cyclophilin D (Du et al., 2008), and OXPHOS complexes including Complex IV (Hernandez-Zimbron et al., 2012) and ATP synthase (Beck SJ. et al., 2016). Furthermore, a pronounced feature of AD pathogenesis is the mitochondrial accumulation of Aβ (Caspersen et al., 2005; Manczak et al., 2006; Cha et al., 2012). Aβ_42_ has been shown to enter mitochondria through the TOM complex, associating with the MIM fraction in a Δψ_m_-independent manner and localizing to cristae in both *in vitro* import systems and in mitochondria from human brain biopsies (Hansson Petersen et al., 2008). The specific interactions of Aβ with the TOM complex were explored using a yeast mitochondria model system, indicating that Aβ binds directly to the Tom22 receptor, but not Tom20 or Tom70, and that Aβ residues 25–42 were indispensable for this interaction (Hu et al., 2018). The accumulation of Aβ within the import machinery is functionally relevant because it inhibits the import of native nuclear-encoded proteins (Sirk et al., 2007). However, it should also be noted that cytosolic Aβ may inhibit the import of mitochondrial proteins by coaggregation in the cytosol that does not involve blocking the mitochondrial import machinery (Cenini et al., 2016). A common theme in these studies is that the more aggregation-prone Aβ_42_ has a stronger interaction with the import machinery than the Aβ_40_ variant (Sirk et al., 2007; Cenini et al., 2016; Hu et al., 2018). Figure 10A (*right*) summarizes the interactions of Aβ with mitochondria.

As mentioned, ApoE4, the primary genetic risk factor for AD, induces increased connectivity between MAMs and mitochondria in different AD models (Area-Gomez et al., 2012; Tambini et al., 2016). MAMs have lipid raft-like membranes, enriched in sphingolipids and cholesterol, which promote the interaction between APP and its processing enzymes (β-secretase and γ-secretase) that have been found to localize at the MAM. The biophysical properties of these microdomains may enhance amyloidogenic processing of APP (Diaz et al., 2015; Del Prete et al., 2017; Area-Gomez et al., 2018). Additionally, the protein interactome of MAMs in cells with mutant APP is enriched in mitochondrial import components Tom22, Tim17b, and Sam50 (Del Prete et al., 2017). Enhanced proximity of APP to the mitochondrial import machinery could contribute to spurious import of APP.

There are some potentially confounding factors regarding the import of the Aβ peptide through the mitochondrial import machinery. The first is that Aβ produced from APP proteolysis is released into exocytoplasmic compartments (ER lumen or the extracellular space), whereas engagement with the mitochondrial import machinery occurs from the cytosol. However, there is evidence that proteolytically produced or externally added Aβ can be taken up by cells via clathrin-dependent or -independent endocytosis, followed by endosomal escape, which could allow it to localize to mitochondria (Saavedra et al., 2007; Hansson Petersen et al., 2008; Berridge, 2010; Friedrich et al., 2010). Furthermore, Aβ produced in the vicinity of mitochondria by MAM-localized secretases could directly access mitochondria (Area-Gomez et al., 2009). The second is that the presence of Aβ in the matrix would necessitate TIM23-mediated translocation; however, to date there is no evidence of a direct interaction between Aβ and the TIM23 complex. One explanation could be that the association between the short Aβ peptide and the TIM23 receptors and channel is too transient to be captured by techniques like crosslinking or immunoprecipitation. Lastly, the Aβ sequence does not contain any segments with strong amphipathicity (opposing basic and nonpolar faces) that are present in canonical MTSs (Figure 9Bii). This feature may hinder transport along the “acid chain” of the TIM23 pathway, possibly explaining how Aβ could stall nonproductively at the TOM complex.

It is noteworthy that AD is associated with a general disruption of the mitochondrial import machinery. Analysis of RNA-seq datasets from brains of AD patients versus age-matched healthy individuals showed that expression of mitochondrial import genes was decreased with AD (Sorrentino et al., 2017), consistent with the observed decrease in expression of Tom20 and Tom70 in postmortem neocortex samples of AD patients that correlate with higher Aβ_42_/Aβ_40_ ratios (Chai et al., 2018). It has also been proposed that the length of a poly-T polymorphism in the *TOMM40* gene correlates with LOAD, supporting the involvement of the TOM complex channel in AD pathogenesis (Roses et al., 2010); however, this finding has been questioned (Chiba-Falek et al., 2018). It is possible that altered expression of TOM complex subunits could modulate mitochondrial interactions of APP/Aβ; for instance, decreased Tom20/70 expression relative to Tom22 could promote Aβ binding to the TOM complex (Hu et al., 2018).

The most compelling evidence that the mitochondrial import and processing machinery serves as a mechanism for amyloid clearance comes from the interaction between Aβ and PreP, the matrix-localized protease that digests MTSs (Pinho et al., 2014). This degradation step is critical because MTSs, being amphipathic and membrane-interactive, can disrupt MIM integrity and their buildup can lead to Δψ_m_ collapse and OXPHOS uncoupling (Teixeira and Glaser, 2013). Importantly, PreP was shown to degrade Aβ variants *in vitro* (Falkevall et al., 2006) and PreP activity is lower in the temporal lobes of AD patients and transgenic AD mice compared with controls, which may be further attributed to disease-related oxidative modifications (Alikhani et al., 2011b). Given the functional coupling between precursor processing and MTS turnover (Kucukkose et al., 2021), the accumulation of Aβ could overcome the MTS-clearing capacity of PreP and cause feedback inhibition of MIP and MPP, thereby inhibiting import by the toxic buildup of precursors and MTSs in the matrix (Mossmann et al., 2014). Conversely, overexpression of PreP in AD mouse models decreases mitochondrial Aβ concentrations and improves organellar function (Fang et al., 2015; Brunetti et al., 2016; Du et al., 2021). Given the key role of PreP in proteostasis (Brunetti et al., 2021), mitochondrial import of Aβ and its subsequent degradation may be essential for balancing cellular Aβ concentrations.

### 5.3 Interactions of α-syn with the mitochondrial import machinery

Several studies support the entry of α-syn into mitochondria through the import complexes. α-syn was first shown to accumulate in mitochondria in a manner that required the Δψ_m_ across the MIM and an accessible Tom40 channel (Devi et al., 2008). Furthermore, the possibility that the N-terminus of α-syn targets mitochondria is supported by the indispensable role of residues 1–32 in mitochondrial uptake (Devi et al., 2008) and of residues 1–11 in association of α-syn with isolated mitochondria (Robotta et al., 2014). When folded as an α-helix (stabilized by membrane interactions), the N-terminal 32 residues of α-syn form an amphipathic structure (Figure 9Biii), made up in part by the first two imperfect KTKEGV repeats that produce a Lys-rich basic face and a Val-rich nonpolar face. The N-terminus of α-syn therefore has strong potential to act as a cryptic MTS (Devi and Anandatheerthavarada, 2010).

TIM23-mediated uptake of α-syn is also supported by its physical and genetic interactions with subunits of the import machinery. Based on proteomics analysis of the interactome of the α-syn C-terminal peptide and its variants phosphorylated at Tyr125 and Ser129 that are known to promote aggregation (Samuel et al., 2016; Fayyad et al., 2020), the unmodified and phosphorylated peptides were found to preferentially interact with mitochondrial and cytosolic proteins, respectively (McFarland et al., 2008). In this study, the unmodified α-syn C-terminal peptide bound Tom40, Sam50, and Tom22, with the interaction to MOM proteins Tom40 and Sam50 strongly reduced by phosphorylation, suggesting the preferential import of unmodified α-syn (McFarland et al., 2008). This connection between α-syn aggregation propensity and mitochondrial import is further supported by the fact that all known PD-related missense mutations reside in the N-terminal region (Flagmeier et al., 2016) near the cryptic targeting sequence (Figure 7A). Perhaps more compelling are the specific ways in which N-terminal mutants of α-syn feature altered cellular interactions; namely, the A53T mutant shows increased affinity for mitochondria and the MAM, whereas the A30P mutant shows weaker affinity (Cole et al., 2008; Devi et al., 2008; Guardia-Laguarta et al., 2014; Pozo Devoto et al., 2017). This may be related to the decreased binding affinity of A30P mutant α-syn for lipid membranes (Jo et al., 2002; Fortin et al., 2004). Therefore, these PTMs and site mutations likely alter the aggregation propensity and membrane interactions of α-syn that subsequently affect its interaction with the mitochondrial import machinery in complex ways.

The involvement of the TOM complex in α-syn import was further investigated in two other studies. In one report, brain tissue taken from postmortem PD patients and transgenic mice overexpressing α-syn showed decreased expression of Tom40 concurrent with mtDNA damage, oxidative stress, and reduced bioenergetic efficiency (Bender et al., 2013). Another report used a rotenone-induced Complex I dysfunction model of PD, as well as postmortem brain tissue of PD patients, to show that α-syn (specifically, the S129 phosphomimetic and soluble oligomers) demonstrated strong but reversible binding to Tom20 and reduced Tom20 expression. This binding thereby prevented interactions between the Tom20/Tom22 receptors, inhibited the import of mitochondrial proteins, and inhibited respiration (Di Maio et al., 2016b). In these studies, the overexpression of either Tom40 (Bender et al., 2013) or Tom20 (Di Maio et al., 2016b; De Miranda et al., 2020) reversed α-syn aggregation and its associated mitochondrial defects. Taken together, these results show a direct interaction between α-syn and the TOM complex, although exactly how the oligomeric state of α-syn or its PTMs may modify such interactions remain open questions. It should be noted that to date, there is no strong evidence for the specific interaction between α-syn and the TIM23 import complex of the MIM. However, transport and sorting of α-syn via the TIM23 machinery is highly likely given the submitochondrial distribution of α-syn, which includes the MIM and matrix. Figure 10B summarizes the interactions of α-syn with mitochondria.

Another feature supporting the mitochondria-targeting capacity of the α-syn N-terminus is its membrane-interactive nature. In its functional role of fusing synaptic vesicles with the presynaptic membrane, α-syn binds anionic phospholipid bilayers through its N-terminus, which then adopts a stable α-helical structure and displaces the unstructured C-terminal end resulting in an elongated conformation (Bartels et al., 2010). Similar membrane interactions have long been known for *bona fide* mitochondrial MTSs, which can undergo a coil-to-helix transition upon anchoring to lipid bilayers without perturbing membrane integrity (Skerjanc et al., 1987; Hoyt et al., 1991; Wieprecht et al., 2000). Functionally, this could allow the mitochondrial precursor to undergo a two-dimensional random walk on the membrane surface to seek out its cognate import receptor more efficiently. It is possible that α-syn adopts a similar mechanism to accumulate on membrane surfaces to increase its local concentration in the vicinity of import complexes.

The interaction of α-syn with the mitochondrial import machinery has important mechanistic implications for mitochondrial stress responses and quality control related to PD (Thorne and Tumbarello, 2022). One of the main pathways for mitophagy-based removal of damaged mitochondria is the PINK1/Parkin system. Under non-stressed conditions, the serine/threonine kinase PINK1 engages mitochondrial import complexes and becomes proteolyzed and efficiently degraded; however, under stress conditions that lower the Δψ_m_ of the MIM, PINK1 accumulates at the MOM and recruits the E3 ubiquitin ligase Parkin, which ubiquitinylates mitochondrial proteins. This signals for autophagic degradation of dysfunctional organelles and consequently blunts cellular expansion of the damage (Ge et al., 2020). Importantly, defects in PINK1 and Parkin (encoded by *PINK1* and *PRKN* genes, respectively) cause the deregulation of mitochondrial quality control and together they represent the preeminent monogenic forms of heritable PD (Klein and Westenberger, 2012).

Moreover, the specific affinity of the α-syn N-terminal region for cardiolipin (Ramakrishnan et al., 2003; Cole et al., 2008; Nakamura et al., 2008; Grey et al., 2011; Robotta et al., 2014) may explain its interaction with mitochondria during stress. The translocation of cardiolipin from the MIM to the MOM is an early signaling event in mitophagy and apoptosis (Li et al., 2015), and it has been shown using α-syn mutant models of PD that cardiolipin becomes externalized to the cytosol, where it then binds α-syn and promotes the refolding of α-syn monomers from aggregated fibrils (Ryan et al., 2018). This may represent a feed-forward process whereby the stress of α-syn burden causes cardiolipin externalization, thereby resulting in the recruitment of more α-syn to the mitochondrial surface. Mitophagy then results when α-syn burden outmatches the refolding ability of cardiolipin.

It should be emphasized that α-syn may play a physiological role in mitochondria (Faustini et al., 2017). For example, consistent with the general role that α-syn appears to play in cellular lipid metabolism and signaling (Jenco et al., 1998; Golovko et al., 2005; Narayanan et al., 2005; Golovko et al., 2006), the lack of α-syn in *SNCA* ^-/-^ mice causes mitochondria to have reduced cardiolipin with altered acyl compositions (Ellis et al., 2005; Barcelo-Coblijn et al., 2007). This effect on cardiolipin in turn alters the physical properties of the MIM coupled with reduced Complex I and III activity (Ellis et al., 2005) and is accompanied by increases in neutral lipids including cholesterol and cholesterol esters (Barcelo-Coblijn et al., 2007). Other studies indicate that α-syn may play critical roles in mitochondrial dynamics, quality control and transport (reviewed in (Pozo Devoto and Falzone, 2017)). While these roles of α-syn could in principle be exerted within or outside the mitochondrion, some reports indicate a role of α-syn within the organelle. For example, α-syn appears to interact directly with the matrix-facing catalytic domain of mitochondrial ATP synthase, with monomers positively regulating its catalytic activity (Ludtmann et al., 2016) and oligomers promoting the mitochondria permeability transition involved in cell death (Ludtmann et al., 2018). This functional duality indicates that, as with cytoplasmic α-syn, it may be the abundance and/or aggregation of α-syn within the mitochondrion and not the mere presence of α-syn itself, that dictates pathogenicity inside the organelle. As such, the TIM23 machinery must then play a key role in regulating this balance of mitochondrial α-syn.

### 5.4 Interactions of Htt/mHtt with the mitochondrial import machinery

In Htt, the site of pathogenic poly-Q expansion is flanked by the first 17 residues of the N-terminus (Nt17) and the proline-rich domain (PRD) (Figure 8A). The Nt17 sequence has the hallmarks of a moderately amphipathic MTS (Figure 9Biv). Many structural and computational studies have addressed the conformational dynamics of the Nt17 sequence in the context of the tripartite structure of Htt. Nt17 itself does not adopt a stable secondary structure but has the characteristics of a compact coil (Thakur et al., 2009), which is similar to other IDPs (Uversky, 2002), and has the propensity to form α-helical structures, particularly in the presence of membranes (Davies et al., 1997; Tam et al., 2009). Indeed, Nt17 forms α-helices in the context of oligomers or fibrils of Httex1 fragments (Kim et al., 2009; Sivanandam et al., 2011; Michalek et al., 2013). The α-helical Nt17 structure promotes aggregation of Httex1 by promoting helical structure within the poly-Q tract (Thakur et al., 2009). By contrast, the PRD forms a polyproline II structure (PPII), which reduces aggregation propensity (Bhattacharyya et al., 2006). Importantly, Htt constructs lacking the Nt17 fail to localize to mitochondria, supporting a role of this segment in mitochondrial targeting (Rockabrand et al., 2007).

Using a combination of biochemical and microscopy-based analyses with striatum-derived cell lines and murine models of HD, Friedlander and colleagues found that Htt and mHtt (and N-terminal fragments thereof) interact directly with the mitochondrial TIM23 complex and are localized to the IMS (Yano et al., 2014; Yablonska et al., 2019) (Figure 10C). In these studies, immunoprecipitation/mass spectrometry and surface plasmon resonance spectroscopy were used to show interactions with TIM23 complex subunits Tim23, Tim50 and Tim17a. They revealed that mHtt had higher affinity interactions with TIM23 subunits and inhibited the TIM23-mediated import of native mitochondrial proteins to a greater extent than wild type Htt counterparts. Importantly, these studies showed that both the Nt17 segment and the expanded poly-Q tract are crucial for interactions with the TIM23 complex. The authors concluded that this inhibition of mitochondrial import by mHtt is an early event in HD pathology (detected pre-symptomatically) and that this effect alone can result in neuronal cell death. Figure 10C summarizes the interactions of mHtt with the mitochondrial import machinery.

These observations provide potential clues as to how Htt, specifically N-terminal segments of mHtt, may engage and disrupt the TIM23 machinery during HD-related proteostatic stress. First, the requirement of Nt17 for TIM23 interactions suggests that this sequence acts like a MTS, assuming an amphipathic α-helical structure to engage the TOM/TIM23 receptors and accumulate Htt/mHtt at mitochondria during HD progression. Second, because TIM23 substrates must be unfolded to traverse the import pathway, N-terminal fragments of Htt/mHtt may be more likely to engage TIM23 than full-length Htt/mHtt, as this would require significant unfolding for import. Finally, the selective interaction of mHtt fragments with TIM23 suggests that the poly-Q expansion provides a sufficiently long, unstructured tether for the Nt17 to access the TIM23 binding sites. Indeed, it has been shown that a sufficiently long presequence is required for TIM23 precursors to span both mitochondrial membranes and engage matrix mtHsp70, which translocates substrates by an active power stroke or Brownian ratchet mechanism (Matouschek et al., 1997; Okamoto et al., 2002). The localization of Htt/mHtt to the IMS suggests incomplete translocation of these polypeptides into the matrix, creating stalled translocation intermediates that could explain the inhibition of TIM23 import. It should be noted that the findings of the Friedlander group were subsequently questioned in a study that found mitochondria-localized mHtt to reside only on the cytosolic side of the MOM and to have no measurable effect on the import of TIM23 substrates (Hamilton et al., 2020). Future work will be required to reconcile these contradictory findings.

## 6 Discussion

We have reviewed current evidence that APP/Aβ, α-syn, and Htt/mHtt interact with the mitochondrial import machinery through their cryptic N-terminal targeting sequences. These interactions have implications for the physiological roles of these proteins as well as their pathogenic interactions in AD, PD, and HD, respectively. They also set the stage for addressing how mitochondrial import may serve as a clearance mechanism for amyloids during proteostatic stress, as well as presenting new directions for developing interventions for neurodegenerative diseases. These two questions are addressed below.

### 6.1 Proteostatic mechanisms that may involve mitochondrial import

**FIGURE 11:**
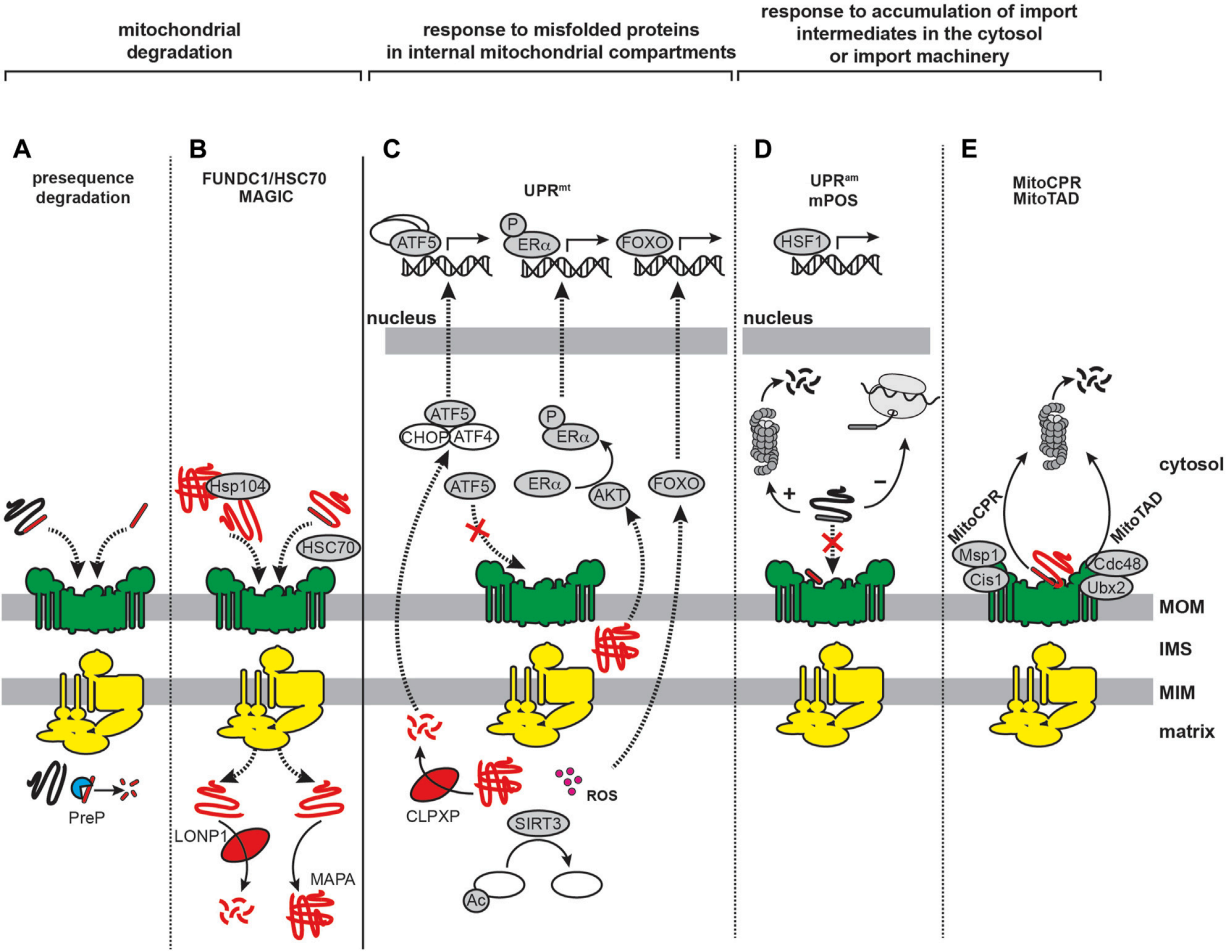
Mitochondrial proteostatic stress responses involving the import machinery. Depicted are complementary stress responses of mammalian systems that involve the mitochondrial import machinery and may be upregulated with amyloid burden. **(A)** PreP degradation. The PreP peptidase that cleaves MTSs also degrades Aβ in the matrix and may play a broader role in clearing imported peptides, including amyloids. **(B)** MAGIC and FUNDC1/HSC70. By these processes, cytosolic proteins are imported into mitochondria for sequestration and proteolysis. In the MAGIC pathway, cytosolic Hsp104 dissociates aggregated proteins, which are imported via the TOM complex into the matrix, where they are degraded by the Pim1 (yeast)/LONP1 (human) protease. In the FUNDC1/HSC70 axis, the MOM protein FUNDC1 interacts with cytosolic HSC70 to import unfolded proteins that are either degraded by LONP1 or assembled into non-aggresomal mitochondrion-associated protein aggregates (MAPAs) that can be subsequently degraded by autophagy. **(C)** UPR^mt^. This process involves transcriptional reprogramming based on mitochondria-nuclear communication in response to protein aggregates in the matrix. The UPR^mt^ involves multiple pathways (1). The ATF5 transcription factor is imported into mitochondria under non-stressed conditions, but when its import is hindered it traffics to the nucleus with other transcription factors (ATF4/CHOP) to activate genes encoding mitochondrial chaperones and proteases. Peptides from the degradation of misfolded mitochondrial proteins, likely produced by CLPXP, may then be exported to facilitate ATF5 translocation (2). Protein aggregates in the IMS activate the AKT kinase, which phosphorylates ERα, serving as a transcription factor to induce expression of IMS-specific proteases (3). Matrix aggregates and ROS activate the sirtuin SIRT3 to deacetylate matrix proteins and activate the FOXO transcription factor that activates mitochondrial antioxidant genes. **(D)** UPR^am^ and mPOS. These reactions respond to cytotoxic accumulation of mitochondrial precursor proteins in the cytosol resulting from defective import. The response of UPR^am^ includes activation of the proteasome, whereas that of the mPOS pathway involves global reduction in cytosolic protein synthesis. Upregulation of the HSP1 transcription factor increases expression of chaperones and other stress response factors. **(E)** MitoTAD and MitoCPR. These systems remove stalled, nonproductive intermediates from the import machinery by continual monitoring of the TOM complex. TOM complex-associated adaptor proteins Cis1/Ubx2 recruit AAA ATPases Msp1/Cdc48 to extract stalled precursors and direct them to the proteasome for degradation.

Mitochondrial proteostasis involves many stress response pathways that either directly or indirectly involve the protein import machinery (Figure 11), and any combination of them could be marshalled in response to amyloid burden at the TIM23 complex. First, some responses involve the degradation of toxic peptides or proteins in the matrix subcompartment. As noted above, the MTS-degrading enzyme PreP (Figure 11A) has an established role in the breakdown of Aβ (Falkevall et al., 2006) and may assume a more general role in the clearance of other amyloidogenic peptides. Other pathways have been described whereby mitochondria import unfolded or aggregated proteins into the matrix for degradation, providing a proteostatic mechanism for the clearance of cytotoxic proteins. These mechanisms, termed Mitochondria as Guardian in Cytosol (MAGIC) (Ruan et al., 2017) and the FUNDC1/HSC70 pathway (Li Y. et al., 2019), both entail the uptake of aggregation-prone proteins into mitochondria with subsequent degradation by matrix proteases. Thus, these pathways may explain why some amyloidogenic proteins are ectopically imported into mitochondria (Figure 11B). Alternative quality control pathways involve feedback mechanisms between the mitochondria and the nucleus to signal defects in mitochondrial protein biogenesis. Among them, the best characterized is the mitochondrial unfolded protein response (UPR^mt^), which senses the accumulation of unfolded proteins and ROS in mitochondrial compartments and activates transcription factors to express nuclear genes encoding mitochondrial chaperones, proteases, and antioxidant systems (Wodrich et al., 2022) (Figure 11C). Indeed, UPR^mt^ activation is implicated in neurodegeneration (Zhu et al., 2021), and particularly in AD (Beck JS. et al., 2016; Sorrentino et al., 2017; Shen Y. et al., 2019), PD (Cooper et al., 2017), and HD (Berendzen et al., 2016; Fu et al., 2019). Other stress responses serve to clear the cytosol of mitochondrial precursor proteins when import is compromised. Namely, the UPR activated by the mistargeting of proteins (UPR^am^) (Wrobel et al., 2015) and the associated mitochondrial precursor overaccumulation stress (mPOS) response (Wang and Chen, 2015) (Figure 11D) respond to increased cytosolic concentrations of precursors, which are normally very low, by activating the proteasome and downregulating cytosolic protein synthesis, respectively. Other similar responses are designed to extract stalled translocation intermediates from the TOM complex and direct them to the proteasome for degradation. These include the mitochondrial compromised import response (MitoCPR) system (Weidberg and Amon, 2018) and the mitochondrial protein translocation-associated degradation (MitoTAD) system (Martensson et al., 2019) (Figure 11E). Although many of these stress responses have been resolved in lower eukaryotes, there is evidence that they exist in mammalian systems as well, although the mechanistic details have yet to be elucidated.

One confounding factor in these mitochondria-based proteostasis mechanisms is the question of how aggregated proteins in the cytosol could access the TOM/TIM23 machinery, given that the import requires an unfolded precursor. A potential solution may come from the observation that a cytosolic HSP70/co-chaperone system is capable of disaggregating amyloid structures (Wentink et al., 2020). Such a system could in principle dislodge amyloid proteins and deliver them as monomers in a post-translational, chaperone-bound fashion to the mitochondrial import machinery. Indeed, several amyloids are found as distinct disease-associated polymorph structures (Qiang et al., 2017; Yang et al., 2022) that could differ in their stabilities and proclivities to dissociate soluble species. A second possibility is that since amyloid fibril structures feature rigid cores surrounded by disordered “fuzzy coats”, the solvent exposure of these flexible regions could promote PTMs and/or dissociation of monomers (Lin et al., 2017). Thus, shed monomers or proteolytic fragments of amyloidogenic proteins from fibrillar aggregates could then be free to interact with the mitochondrial protein machinery.

In summary, the degree to which PreP, MAGIC, and FUNDC1/HSC70 may assist as general clearance systems for amyloidogenic proteins like Aβ, α-syn, or mHtt requires further investigation, as do the pathways by which these amyloids may trigger mitochondrial import-related stress responses.

### 6.2 Prospects for drug development

The interactions between amyloidogenic proteins and the mitochondrial import machinery may inform strategies for developing novel therapeutic interventions for neurodegenerative diseases. Clinical studies and drug discovery efforts have led researchers to question the role of amyloid fibrils themselves as the primary causative agents in AD, PD, and HD. For example, the extent of fibril formation does not always correlate with disease severity (Breydo et al., 2012; Ow and Dunstan, 2014), fibrils can be present even in the absence of disease symptoms (Ke et al., 2020), and therapeutic strategies designed to disrupt fibril formation have been met with mixed success (Pardridge, 2016; Barker and Mason, 2019; Paolini Paoletti et al., 2020; Pardridge, 2020; Yiannopoulou and Papageorgiou, 2020). Hence, alternative intervention approaches, including targeting mitochondrial dysfunction, are being developed for neurodegenerative disorders (Xu et al., 2022b). Here we review some promising interventions that directly or indirectly involve the amyloid-TIM23 pathway interaction.

From a broad perspective, given that AD, PD, and HD are all diseases of aging (Blumenthal, 2004; Taylor and Dillin, 2011), targeting general aging-related mitochondrial dysfunction may be an effective strategy. For example, the efficiency of the UPR^mt^ declines with age (Bennett and Kaeberlein, 2014; Munoz-Carvajal and Sanhueza, 2020) making it a potential target for neurodegenerative diseases (Suarez-Rivero et al., 2022). Indeed, compounds that increase cellular NAD^+^ levels, including nicotinamide riboside and olaparib, improved longevity in *C. elegans* models of Aβ proteotoxicity by activation of UPR^mt^ and mitophagy (Sorrentino et al., 2017). Because the UPR^mt^ involves upregulation of the TIM23 machinery (Xin et al., 2022), mitochondrial import may either be an important target of UPR^mt^-modulating compounds, or serve as a marker for pharmacological UPR^mt^ activation. Another mitochondrial parameter that declines with age is the Δψ_m_ (Sugrue and Tatton, 2001; Berry and Kaeberlein, 2021; Berry et al., 2023). Caloric restriction (CR) and rapamycin, which extend lifespan in several wild type organisms (Nakagawa et al., 2012; Bitto et al., 2016; Shindyapina et al., 2022), can mediate their effects by regulating Δψ_m_ (Paglin et al., 2005; Cheema et al., 2021; Berry et al., 2023). These treatments could therefore operate in part by rescuing TIM23-mediated protein import, which functions in a Δψ_m_-dependent manner. However, thorough pre-clinical testing of these interventions in AD, PD, and HD models would be warranted as CR and UPR^mt^ activation can shorten or extend lifespan depending on the context in which the treatments are applied (Liao et al., 2010; Bennett and Kaeberlein, 2014; Angeli et al., 2021; Xin et al., 2022). Notably, a recent study with rapamycin in a mouse model of AD showed increased amyloid plaque formation upon treatment (Shi et al., 2022).

Given the potential role of mitochondria-resident proteases in amyloid degradation, these may represent pharmacological targets. For instance, as mentioned, the mitochondrial PreP enzyme degrades MTSs and has been implicated in degradation of Aβ in the matrix (Falkevall et al., 2006; Alikhani et al., 2011b; Mossmann et al., 2014; Fang et al., 2015; Du et al., 2021); thus, PreP may represent a potential drug target for neurodegeneration (Brunetti et al., 2021). In fact, researchers using the Senescence Accelerated Mouse Prone 8 (SAMP8) AD model found that restoring global mitochondrial function with metabolic modulators (essential and branched-chain amino acids) was accompanied by a reversal of aging-related declines in PreP levels (Brunetti et al., 2020). Similarly, researchers using the APPswe/PS1dE9 AD model, associated with FAD mutations in APP and γ-secretase, found that treatment with the neuroprotective compound Ligustilide reduced disease progression with a concurrent increase in PreP levels (Xu et al., 2018). Very recently, Pioglitazone, an antagonist of the PPAR-γ transcription factor that regulates mitochondria structure and function, was shown to upregulate PreP and the insulin degrading enzyme IDE, thereby restoring the peptide processing machinery (Di Donfrancesco et al., 2023). Original efforts toward developing agonists of PreP were focused on small molecule benzimidiazole derivatives (Vangavaragu et al., 2014), but these results were called into question (Li NS. et al., 2019). Therefore, the potential efficacy of specific effectors of PreP requires further validation.

Finally, there are several therapeutic compounds effective in treating neurodegenerative diseases that improve mitochondrial function as part of their mechanism of action. Three such compounds, described here, could have direct or indirect effects on mitochondrial protein import. First, the synthetic tetrapeptide SS-31 (elamipretide) targets mitochondria by interacting with the cardiolipin-rich MIM (Szeto, 2014). SS-31 is protective against mitochondrial dysfunction associated with a range of diseases, including myopathy, cardiac, retinal, and kidney diseases, and has demonstrated neuroprotective function (Zhu et al., 2018; Zhao et al., 2019; Liu Y. et al., 2021; Nhu et al., 2021), including efficacy in models of AD (Manczak et al., 2010; Calkins et al., 2011; Reddy et al., 2017; Reddy et al., 2018), PD (Yang et al., 2009), and HD (Yin et al., 2016). In these studies, SS-31 has been shown to restore mitochondrial biogenesis, dynamics and energetic output, reduce oxidative stress, and preserve mitochondria structure, with a notable effect on upregulating TOM complex receptor expression (Reddy et al., 2018). SS-31 and its side chain variants may act by modulating membrane electrostatics (Mitchell et al., 2020; Mitchell et al., 2022), mitigating Ca^2+^ stress at the MIM (Mitchell et al., 2020), and/or preserving the Δψ_m_ (Zhang et al., 2020). Furthermore, SS-31 has an extensive interactome in mitochondria (Chavez et al., 2020) and may therefore attenuate pathogenic interactions of peptides such as Aβ and α-syn with different mitochondrial proteins. Second, the curcumin derivative J147 is a neuroprotective compound that has been explored as an effective treatment in AD models (Prior et al., 2013; Goldberg et al., 2018; Currais et al., 2019; Goldberg et al., 2020; Kepchia et al., 2021; Kepchia et al., 2022). Based on these studies, several molecular mechanisms have been ascribed to J147, including improved energy metabolism, modulation of Ca^2+^ flux and activation of the AMPK/mTOR pathway, reduction of plasma free fatty acid levels, and regulation of acetyl-CoA metabolism. Third, polyphenols constitute a broad class of phytochemicals, many of which show neuroprotective properties (Naoi et al., 2019). Among them, urolithin A has demonstrated efficacy in models of AD (Ballesteros-Alvarez et al., 2023) and PD (Liu et al., 2022). Mechanistically, urolithin A activates mitophagy (Ryu et al., 2016; Amico et al., 2021), which may reverse defects in PINK1/Parkin mitophagy that are implicated in both familial and sporadic PD (Dawson and Dawson, 2010). Because the PINK1/Parkin mechanism is based on interactions with the TIM23 pathway, urolithin A may help reduce proteostatic stress caused by interactions of α-syn with the import machinery. Notably, SS-31, J147, and polyphenolics are all known to interact with mitochondrial F_1_F_O_ ATP synthase (Ahmad and Laughlin, 2010; Goldberg et al., 2018; Chavez et al., 2020), an enzyme whose dysfunction is implicated in neurodegeneration, particularly AD (Ebanks et al., 2020). Increasing evidence supports that dysfunction of ATP synthase coincides with AD progression (Liang et al., 2008; Terni et al., 2010; Beck SJ. et al., 2016), which may mechanistically occur by interactions of Aβ with the OSCP subunit (Beck SJ. et al., 2016). It has also been shown that loss of OSCP drives the opening of the mPTP and activates the UPR^mt^ (Angeli et al., 2021). Hence, compounds that bind ATP synthase may help maintain its function during amyloidogenic stress and/or inhibit mPTP pore opening induced by the pore initiator cyclophilin D, producing a result similar to the action of cyclosporin A (Connern and Halestrap, 1994; Gauba et al., 2019). Such interventions could increase ATP synthase activity and prevent membrane depolarization, thereby improving available ATP levels to enhance proteostatic clearance and matrix-directed import through the TIM23 pathway.

## 7 Conclusion

Aβ/APP, α-syn, and Htt/mHtt all contain MTS-like sequences that promote their interactions with the mitochondrial TIM23 import machinery. These interactions can directly impact mitochondrial protein import function, as well as allow incorporation of amyloids into mitochondrial subcompartments, which could contribute to pathogenicity in AD, PD, and HD, respectively. The engagement of Aβ/APP, α-syn, and Htt/mHtt with the import complexes may be part of the normal physiological function of these proteins, may represent an amyloid clearance mechanism, or may be purely pathogenic. Addressing these questions will be critical in understanding the role of mitochondria in neurodegeneration. We note that evidence for the engagement of amyloids with the import machinery does not necessarily favor either the amyloid or mitochondrial cascade hypotheses, as these interactions could occur under both scenarios. However, determining whether the amyloid-import machinery interaction is an upstream cause, or a downstream effect will help resolve the role of mitochondrial dysfunction in the sequence of events associated with neurodegeneration. Further investigation of the interactions of amyloidogenic proteins with the mitochondrial import machinery will add to our understanding of the role of mitochondria in proteostatic stress and facilitate the development of therapeutic interventions.
