# Supplementary material for: Interactions of amyloidogenic proteins with mitochondrial protein import machinery in aging-related neurodegenerative diseases
Source: Front Physiol. 2023 Nov 2;14:1263420. doi: 10.3389/fphys.2023.1263420 (PMC10652799; doi:10.3389/fphys.2023.1263420)
Supplement: Supplementary file 1 [file Table1.pdf]

**Supplementary Table 1.** Mitochondrial protein import and quality control components - human and yeast homologs[1]

| | Complex[2] | Human (mammalian)[3] | Fungal homolog[3] | Localization[4] | Function[5] | |
|---|---|---|---|---|---|---|
| Cytosolic targeting factors | | HSP70 | Hsp70 | cytosol | ATP-dependent molecular chaperone | |
| | | HSP90 | Hsp90 | cytosol | ATP-dependent molecular chaperone | |
| | | NAC | NAC | cytosol | Ribosome-binding factor / recruitment of RNCs to mitochondria | |
| | | SYNJ2BP | —— | MOM | Putative mRNA binding factor | |
| TOM and SAM complexes | TOM | TOM40 | Tom40 | MOM | Channel-forming $\beta$-barrel | core complex |
| | | TOM22 | Tom22 | MOM | Receptor / central organizer | |
| | | TOM5 | Tom5 | MOM | Complex assembly / stabilization | |
| | | TOM6 | Tom6 | MOM | Complex assembly / stabilization | |
| | | TOM7 | Tom7 | MOM | Complex assembly / stabilization | |
| | | TOM20 | Tom20 | MOM | Receptor (primarily MTSs) | receptors |
| | | TOM70 | Tom70 | MOM | Receptor (primarily hydrophobic proteins) / chaperone | |
| | SAM | SAM50 | Sam50 | MOM | Core subunit for $\beta$-barrel integration | |
| | | MTX1/3 | Sam37 | MOM | Accessory subunit for $\beta$-barrel insertion / mitochondrial trafficking | |
| | | MTX2 | Sam35 | MOM | Accessory subunit for $\beta$-barrel insertion / mitochondrial trafficking | |
| TIM23 complex | TIM23 | TIM23 | Tim23 | MIM | Voltage-regulated protein-conducting channel | core complex |
| | | TIM17 A/B1/B2 | Tim17 | MIM | Protein-conducting channel | |
| | | TIM50 | Tim50 | MIM | MTS receptor / phosphatase | |
| | | TIM44 | Tim44 | matrix | Peripheral scaffold | TIM23^MOTOR / PAM-specific subunits |
| | | mtHSP70 (Mortalin) | mtHsp70 (Ssc1) | matrix | ATPase motor | |
| | | DNAJC15 / DNAJC19 | Tim14 (Pam18) | MIM | J-type protein cochaperone | |
| | | MAGMAS 1/2 | Tim16 (Pam 16) | MIM | J-type protein cochaperone | |
| | | GrpEL1/2 | Mge1 | matrix | mtHsp70 nucleotide exchange | |
| | | —— | Pam17 | MIM | Complex organization | TIM23^SORT-specific subunits |
| | | TIM21 | Tim21 | MIM | Sorting of MIM proteins | |
| | | ROMO1 | Mgr2 | MIM | Lateral integration of MIM proteins | |
| Mitochondrial processing peptidases | MPP | PMPCA | Mas2 | matri | Metalloprotease; cleavage of MTSs from matrix-directed precursors | |
| | | PMPCB | Mas1 | | | |
| | IMP | IMMP1L | Imp1 | MIM | Serine protease; cleavage of hydrophobic sorting signals from IMS-directed precursors | |
| | | IMMP2L | Imp2 | | | |
| | | MIP | Oct1 | matrix | Metalloproteases; N-terminal processing of imported proteins after MPP cleavage | |
| | | XPNPEP3 | Icp55 | | | |
| | | PREP | PreP | matrix | Metalloprotease; digestion of presequences | |
| Mitochondrial folding coplexes | Chaperonin | HSP60 | Hsp60 | matrix | Forms enclosed barrel structure with central cavity | |
| | | HSP10 | Hsp10 | | Coordination of ATPase chaperone ATPase activity | |
| | HSP70 | mtHSP70 (Mortalin) | mtHsp70 (Ssc1) | matrix | ATPase-driven chaperone | |
| | | GrpEL1/2 | Mge1 | matrix | mtHsp70 nucleotide exchange | |
| | | TID1 | —— | matrix | mtHsp70 J-type cochaperone | |
| | | HEP1 | Hep1 | matrix | mtHsp70 cochaperone | |
| | HSP90 paralog | TRAP1 | —— | | ATP-dependent molecular chaperone; great functional diversity | |
| Mitochondrial proteases | m-AAA | AFG3L2 | Yta12 | MIM | ATP-dependent metalloprotease; catalytic domian faces the matrix | |
| | | SPG7 (Paraplegin) | Yta10 | | | |
| | i-AAA | YME1L | Yme1 | MIM | ATP-dependent metalloprotease; catalytic domian faces the IMS | |
| | CLPXP | CLPP | —— | matrix | ATP-dependent serine protease | |
| | | CLPX | Mcx1 | | | |
| | | LONP | Pim1 | matrix | ATP-dependent serine protease | |
| | | HTRA2 | —— | MIM | Serine protease | |
| | | PARL | Pcp1 | MIM | Serine protease; processing of proteins involved in mitophagy | |
| | | OMA1 | Oma1 | MIM | Stress-activated metalloprotease; processing proteins involved in stress response and dynamics | |

[1]Color coding of major functional groups corresponds to that used in Figure 2.

[2]Common names of homo- or hetero-oligomeric assemblies that operate as a functional machinery.

[3]Column entries are blank where no corresponding human/mammalian or fungal homologs are known.

[4]For integral membrane proteins, this column indicates the membrane in which they are embedded. For soluble or peripheral proteins, this column indicates the aqueous compartment in which they reside, respectively.

[5]Functional features emphasize those of human/mammalian proteins and complexes.
